# Mechanisms of Traumatic Spinal Cord Injury AIS Grade Conversion

**DOI:** 10.1089/neur.2025.0035

**Published:** 2025-06-16

**Authors:** Jesse A. Stokum, Riccardo Serra, Nicole Gorny, Bradley Wilhelmy, Timothy J. Chryssikos, Gary Schwartzbauer, Bizhan Aarabi, Volodymyr Gerzanich, J. Marc Simard

**Affiliations:** ^1^ Department of Neurosurgery, University of Maryland School of Medicine, Baltimore, Maryland, USA.; ^2^ Department of Pathology, University of Maryland School of Medicine, Baltimore, Maryland, USA.; ^3^ Department of Physiology, University of Maryland School of Medicine, Baltimore, Maryland, USA.

**Keywords:** AIS grade conversion, neuronal stunning, programmed cell death, secondary axonal degeneration, secondary injury, traumatic spinal cord injury

## Abstract

Spinal cord injury (SCI) remains a major unsolved problem that permanently impairs the lives of innumerable individuals worldwide. Although advances in the basic, pre-clinical and clinical sciences of SCI hold promise for patients, clinicians may lack a full insight into the relevant cellular and molecular events, and laboratory researchers may underappreciate how cellular and molecular phenomena translate into meaningful functional outcomes. To help bridge these perspectives, we first review the American Spinal Injury Association (ASIA) Impairment Scale (AIS) grade, which is the principal instrument used to gauge clinical outcomes in SCI, and the clinically important concept of AIS grade “conversion” (improvement), which occurs in some but not all patients. We then review underlying mechanisms that contribute to the AIS grade and its conversion, including mechanisms of transient neurological dysfunction (neuronal and axonal “stunning”), mechanisms of secondary cell loss (apoptosis, pyroptosis, and necroptosis), and mechanisms of axonal loss (primary axotomy and secondary axonal degeneration). Finally, we briefly review approaches to clinical management that may ameliorate identified mechanisms of secondary tissue loss and neurological dysfunction following SCI.

## Introduction 

### Spinal cord injury

Spinal cord injury (SCI) is a devastating pathological event resulting from mechanical damage to the spinal cord. The most common etiologies are falls and motor vehicle accidents, although sports accidents and penetrating injuries also contribute.^1^ SCI has a worldwide prevalence of approximately 20.6 million cases with ∼4/100,000 yearly incident cases in the United States.^2^ While epidemiological profiles of SCI vary across countries, in general, the number of male patients exceeds females,^3^ with the average age of injury at 59.2 years.^1^ Notably, over the past few decades, the average patient age and the proportion of SCIs attributable to falls have increased, whereas the proportion of functionally complete to incomplete injury and the number of patients with fracture dislocation have decreased.^4–6^

SCI is a leading cause of long-term disability, responsible for more than 6.2 million years of life lived with disability, a dramatic increase in the last 3 decades.^7^ Long-term sequelae of SCI may include locomotor dysfunction, loss of hand function, respiratory complications, chronic pain, bowel and bladder incontinence, impaired sexual function, impaired kidney function, and psychiatric abnormalities.^8^

SCI also imposes a large economic burden. Direct costs of acute and long-term care vary with level and grade of injury, location of care, and methodology, but, in the United States, they range from $111,780 to $1,156,410.^9^ The indirect cost of SCI is perhaps more alarming, with the average annual cost per patient estimated above $29,000. The lifetime economic impact of a single patient with SCI is between $0.5 and $2.3 million, with younger age of injury contributing to a greater total lifetime economic burden. Employment rate post-SCI decreases by an estimated 52%.^10^ While the cost to society is substantial, much of the economic burden is borne by SCI patients and their families.

### A bench-to-bedside disconnect in SCI research

The current treatment for SCI consists of timely and adequate surgical decompression and critical care with blood pressure augmentation. A large and fast-growing body of clinical research seeks to improve outcomes in SCI. Areas of current interest include the role of early surgical decompression, optimization of surgical timing for adequate decompression, the use of intraoperative imaging including ultrasound, and advanced neurophysiology monitoring including spinal cord perfusion pressure monitoring.^11,12^ Simultaneously, a growing body of basic science research aims to understand and prevent mechanisms of neuron and axon loss after SCI. This work has yielded a list of exciting (albeit many still experimental) therapies, including calpain inhibitors,^13^ sodium-calcium exchanger (NCX)1 inhibitors,^14^ voltage-gated sodium channel (VGSC) inhibitors,^15–17^ sulfonylurea receptor 1–transient receptor potential melastatin 4 (SUR1-TRPM4) inhibitors,^18,19^ cell-based therapies,^20^ novel neurorehabilitation methods,^21^ and innovative techniques involving brain–computer interface.^22^

While these advances hold great promise for patients with SCI, there often exists a disconnect between clinical and basic science research. Clinicians and therapists may not fully appreciate the cellular and molecular events that mediate improvements or constitute impediments to functional recovery following SCI. Similarly, laboratory researchers may not fully appreciate how cellular or molecular phenomena translate into functional outcomes and quality of life. In this review, we seek to remedy this disconnect by bridging these two perspectives. We first discuss how function is commonly quantified in patients with SCI and then correlate these grading scales with the cellular and molecular events that occur after injury.

## Basic Spinal Cord Anatomy

### Gross anatomy

The spinal cord is composed of a grey matter core encased in white matter that forms an “H” or butterfly shape cross-sectionally. The spinal cord is predominantly (∼85%) comprised of white matter.^23^ The spinal cord’s gray matter is comprised of motor and sensory neuron cell bodies, interneurons, unmyelinated axons, and neuroglia organized into the lateral, dorsal, and ventral horns. The lateral horns, present mainly in the thoracic region of the cord, contain preganglionic autonomic neurons, whereas the dorsal horns contain neurons that receive sensory input from the spinal nerves’ dorsal roots and Clarke’s columns that process proprioceptive information. The ventral horns contain cell bodies of motor neurons terminating on skeletal muscle. Grey matter is further defined by its laminar distribution into 10 distinct subgroups based on topography and density.^24^ The white matter exterior of the spinal cord is comprised of mostly myelinated sensory and motor tracts. The ascending sensory tracts include the dorsal columns, spinothalamic, and spinocerebellar tracts, which carry pressure, vibration, pain, temperature, touch, and proprioception information from the body to higher levels of the central nervous system (CNS). Descending tracts relay information related to conscious skeletal muscle control, posture, balance, and muscle tone via the corticospinal, vestibulospinal, rubrospinal, and reticulospinal tracts. In general, sensory tracts are found dorsally, preganglionic visceral motor tracts laterally, and somatic motor tracts in the ventral region of the cord.^25^

Grossly, the spinal cord can be divided into four distinct regions defined by the vertebral exit point of 31 spinal nerves: the cervical, thoracic, lumbar, and sacral regions. The first 7 cervical nerves exit the cord superior to the corresponding vertebrae, whereas the remaining 24 nerves exit inferiorly.^24^ After exiting the cord, spinal nerves within a region merge to form a network called a spinal plexus. The severity, prognosis, and treatment options for SCI are in part determined by the affected region of spinal cord and plexuses.^26^

### Axon anatomy

White matter and axons comprise nearly 85% of the total spinal cord volume in an adult human male.^23^ Therefore, axon physiology and pathology is intimately associated with spinal cord function. Axons are highly specialized subcellular compartments, described previously as a “cell within a cell.” Interestingly, in certain species of wasp, some axons exist independently of their nuclei, which lyze and die during development.^27^ Axon dimensions vary greatly: axon diameters range from 0.1 to 30 microns in vertebrates and axon lengths can be up to 1 m in humans. Larger axonal diameters support higher firing rates at the expense of greater energy and space requirements.^28,29^

Ultrastructurally, axons contain a cytoskeleton comprised of a microtubule and neurofilament core surrounded by an actin cortex.^29^ Microtubules are ∼25 nm thick, polar filaments of variable length comprised of polymerized α/β tubulin heterodimers arranged longitudinally in overlapping bundles. Microtubules serve as a roadway for axonal transport and contribute to structural stability and branching.^30^ Neurofilaments are ∼10 nm thick, nonpolar filaments comprised of polymerized neurofilament hetero-oligomeric subunits and are arranged longitudinally alongside the microtubule bundles. Neurofilaments form an inert hydrogel “solvent” that influences axon diameter by expanding the cross-sectional area.^29,31^ The axon cortex is comprised of periodically spaced F-actin rings interlocked by spectrin cross-linkers that form a “corset” around the cytoskeletal core. The actin cortex serves as a tension buffer that organizes the axon plasmalemma.^30,32–34^

Axons contain an array of organelles. Mitochondria are present in large numbers to support the high axonal energy demand,^35^ since even at rest a human neuron consumes ∼4.7 billion ATP molecules per second.^36^ Axonal mitochondria are regulated by cytosolic calcium and contribute to calcium buffering.^37–39^ Axons also harbor an extensive reticulated network of thin (∼10 µm) smooth endoplasmic reticulum (SER) tubules.^40^ Axonal SER supports the lipid metabolism and lipogenesis required for axon repair and growth.^41^ Axonal SER is also an important calcium store and conducts intra-axonal signaling between the plasmalemma and organelles or between the axon and the neuronal somata.^42–44^ Axons also contain a robust endosomal and peroxisomal system.^30^

The axolemma of myelinated central axons exhibits regular periodicity in its composition. Nodes of Ranvier, where axonal segments are “bare” and not covered with myelin, are interspersed with axonal internodes, where the investing myelin becomes compacted.^45^ In the regions immediately adjacent to the nodes of Ranvier, the surrounding myelin forms paranodal loops, where cytoplasm fills the myelin lamellae.^45^ VGSCs are abundant at nodes of Ranvier,^46^ whereas potassium channels are primarily present at juxtaparanodal regions.^47^

## SCI AIS Grade Conversion

### SCI grading

The most widely used and best validated clinical scoring system to assess SCI and establish prognosis is the *International Standards for Neurological Classification of Spinal Cord Injury (ISNCSCI)* and the American Spinal Injury Association (ASIA) Impairment Scale (AIS).^48^ This grading scale was first published in 1982 and has undergone several revisions.^49^ Strength is quantified by motor scores and sensation (light touch and pinprick) by sensory scores. The ISNCSCI helps to determine the neurological level of injury (NLI), and the AIS grade summarizes the extent or completeness of injury. AIS grade A injuries have no preservation of motor or sensory function in the S4-5 sacral segments, AIS B injuries have preservation of sensation below the NLI including S4-5, AIS C and D have increasingly preserved motor function below the NLI, and AIS E represents a patient with intact gross motor and sensory function. Of note, the NLI itself does not directly influence the AIS grade.

### AIS grade conversion

Patients presenting AIS grade A–D may later recover some degree of motor and sensory function leading to improvement in the AIS grade, a phenomenon referred to as AIS grade conversion.^50^ The precise definition of AIS grade conversion varies in the literature, but here, we use the term to refer to any upward change in AIS grade after the initial presenting assessment.

There are conflicting opinions and data regarding the reliability and stability of AIS grade obtained acutely after SCI. Some have reported early worsening in the AIS grade during the first 72 h after injury, with later (>72 h) AIS grade assessments correlating better with outcome than early (24 h) assessments.^51,52^ Similarly, others have shown that AIS grade assessments captured at very early time points (<4 h) are more likely to exhibit later grade conversion.^53^ However, other investigators have demonstrated and argued that early (<72 h) AIS grade assessments can be reliably correlated with outcome.^54,55^ Notwithstanding the ongoing debate regarding the prognostic utility of early (<72 h) AIS grade assessments, a subset of SCI patients do appear to exhibit AIS grade fluctuations acutely after injury.

Upward AIS grade conversions can also occur at later timepoints. Approximately 25–30% of patients who are initially categorized as AIS grade A group later convert to AIS grade B or better, ∼65–75% of AIS grade B and C patients convert to improved grades, and ∼10% of AIS grade D patients convert to E.^56–58^ Importantly, conversion does not imply complete neurological recovery since generally, when patients exhibit AIS grade conversion, they convert to the next better AIS grade rather than leap multiple grades.^56^ While most (∼50%) upward conversion occurs within the first month, some patients experience conversion throughout the first year after injury, with decreasing likelihood thereafter.^56^

The practical impact of AIS grade conversion upon patient quality of life must be considered. Patients with SCI place particularly high importance on recovery of hand function, walking, and bowel and bladder function.^59,60^ Recovery of these functions depends heavily on the initial AIS grade. For example, patients who are initially AIS grade A are highly unlikely to recover walking, as only 14% of those who exhibit grade conversion from A being able to achieve functional walking.^61^ Furthermore, if walking is achieved in AIS A patients, they are likely to have low thoracic or lumbar injuries, and the walking is likely to be slow and energy intensive.^62,63^ The odds of walking recovery are better in AIS grade B, C, and D patients, rising to 33%, 75%, and 100%, respectively.^61^ Interestingly, while AIS grade conversion indicates improved function, grade conversion does not correlate well with recovery of walking function, indicating that more targeted neurological outcomes measures are necessary.^61,64^ Total motor score is predictive of bowel function, which is more severely impacted in complete versus incomplete injuries.^65^ Completeness of injury and thus AIS grade is also highly correlated with the likelihood of recovery of hand function.^66^

### Clinical factors associated with AIS grade conversion

Various factors have been linked to AIS grade conversion. Broadly, these factors can be categorized as modifiable versus nonmodifiable. Nonmodifiable factors are related to patient characteristics including demographics, injury mechanism, injury morphology, and presenting neurological status. For example, the initial AIS grade is strongly predictive of grade conversion. As discussed above, patients who are AIS grades A and D are less likely to convert than grades B or C, although for different reasons. The low rate of conversion for AIS A is likely due to injury severity, whereas the low rates of AIS D conversion are probably reflective of a “ceiling effect” inherent to the AIS grading scale itself.^56^ Level of injury is also predictive of grade conversion, with lumbar injuries being the most likely to undergo grade conversion, followed by cervical and finally thoracic injuries.^50^ These segmental differences in vulnerability arise in part from differences in blood supply and grey matter composition.^67^ Other nonmodifiable factors include patient age, mechanism of injury (penetrating versus blunt, motor vehicle collision versus ground-level fall), and genetic polymorphisms.^50^ The extent of the sensory zone with partial preservation or the number of levels below the sensory NLI that retain partial sensation is also predictive of conversion, as preservation of more than three levels is linked to a greater likelihood of conversion.^68^

Modifiable factors are of greater interest to the medical community. Blood pressure augmentation and careful avoidance of hypotension are critical components of the acute management of SCI and have been linked with improved outcomes.^69,70^ The AANS/CNS Joint Section on Spine and Peripheral Nerves recommends that the mean arterial pressure be maintained at 85–90 mm Hg for 7 days after injury.^71,72^ Early (<24 h after injury) and adequate surgical decompression has also been linked with improved outcome.^58,73–75^ Additional experimental interventions, including intrathecal pressure monitoring, have been proposed to help guide blood pressure management. Overall, these modifiable factors likely contributed to the improved rates of AIS grade conversion recently observed versus historical controls.^76^

The precise mechanisms responsible for AIS grade conversion are often vaguely defined, with many authors alluding to improved tissue perfusion and reduction of secondary injury. However, this lack of mechanistic detail presents a barrier to the development of novel targeted therapies. Here, we discuss known mechanisms that underlie AIS grade conversion.

## Mechanisms Underlying AIS Grade Conversion

### AIS grade conversion is not due to healing

Many patients and even some practitioners may witness AIS grade conversion and assume that these patients are simply healing from their injury. However, since the CNS mostly lacks regenerative properties, this cannot be the case. Parenthetically, while endogenous repair mechanisms such as axonal sprouting have been proposed to contribute to functional recovery, their role in motor recovery in human SCI remains unclear.^77,78^ Rather, recovery of function after SCI primarily results from axons and neurons that become temporarily silenced following injury and that later recover function. During this period of neuronal silencing or stunning, secondary injury and the resultant permanent loss of additional axons and neurons may occur, lowering the total pool of potentially viable neural elements and limiting functional recovery. Therefore, AIS grade conversion reflects the integrated effects of two phenomena: (i) secondary injury with ongoing loss of neurons and axons and (ii) temporary “stunning” of the anatomically preserved axons and neurons due to conduction block and hyperpolarization, respectively, also referred to as transient neuropraxia and spinal cord concussion. These phenomena and their relevance to AIS grade conversion are discussed in this section.

### Neuronal stunning creates a “fog of war” in SCI

Following SCI, a subpopulation of injured spinal cord axons and neurons becomes temporarily nonfunctional, a phenomenon that we refer to here as “neuronal stunning” but also referred to as transient neuropraxia and spinal cord concussion. Stunned axons and neurons remain viable and are potentially salvageable but cannot support normal signal transduction. Mechanisms of stunning differ between axons and neuronal somata. Temporary axon dysfunction secondary to conduction block has been observed in perilesional axons in animal models of SCI.^79–81^ Several factors contribute to axon conduction block, including demyelination of perilesional axons,^80,82^ secondary axonal degeneration (SAD), a form of delayed axonal loss that is reversible in early phases and results in the formation of axonal spheroid formation,^83^ edema and hemorrhage with subsequent neuroinflammation, generation of nitrous oxide and free radicals, and localized mass effect resulting in impaired tissue perfusion.^84^ Resolution of conduction block is associated with functional improvement.^80^ Temporary neuronal somata dysfunction is primarily due to post-injury hyperpolarization and decreased excitability.^85^ Of note, a related phenomenon is so-called “spinal shock,” which refers to the temporary loss of spinal cord reflexes caudal to the level of injury secondary to the loss of descending synaptic input.^86,87^ Stunned axons can potentially exist both in the T2-weighted MRI hyperintense lesion itself and in the perilesional tissues that appear radiographically normal.

Neuronal stunning is a temporary situation in which the extent of interrupted neurological function may or may not predict the underlying extent of permanent tissue loss. In essence, neuronal stunning creates a “fog of war” in SCI. The phrase “fog of war” was coined by Sir Lonsdale Augustus Hale in 1896 as “the state of ignorance in which commanders frequently find themselves as regards the real strength and position, not only of their foes, but also of their friends.”^88^ Similarly, due to neuronal stunning, after SCI the actual number of viable axons and neurons is hidden and ongoing losses are obscured. Neuronal stunning therefore complicates baseline assessments in SCI clinical trials and may cloud early prognostication. Neuronal stunning is highly relevant to AIS grade conversion in that its resolution can contribute to AIS grade improvement.

### Tissue bridges highlight functional versus anatomical completeness of injury

Importantly, due to neuronal stunning, what may be thought to be a “complete” SCI (with the designation of completeness based upon the initial neurological examination) may turn out to be an incomplete injury that later exhibits partial functional recovery. For this reason, a careful distinction should be made between “*functional* completeness,” where completeness of injury is based upon the neurological examination, and “*anatomical* completeness,” which corresponds to anatomical tissue loss that would preclude neurological recovery. Therefore, an initially *functionally* complete SCI patient may not harbor an *anatomically* complete injury such as a cord transection but rather have stunned white matter with later partial functional recovery as neuronal stunning resolves. Similarly, patients with incomplete neurological deficits may eventually have complete return of function with no underlying neurological tissue loss at all (e.g., transient neuropraxia in the setting of congenitally narrow spinal canal).

The presence of intact tissue bridges on T2-weighted MRI after traumatic SCI is a clear example of intact white matter tracts that are stunned and may potentially recover. As discussed above, stunned axons and axons undergoing secondary degeneration can exist in both T2-weighted MRI hyperintense lesional tissue and the surrounding perilesional tissue. Multiple studies have validated the prognostic value of tissue bridges, showing that for each mm of preserved tissue, patients gained 7.8–12.1% of maximal sensorimotor recovery at 3 months.^89,90^ Here, we describe an index case that illustrates tissue bridges and highlights the difference between anatomical and functional completeness of injury.

**FIG. 1.**
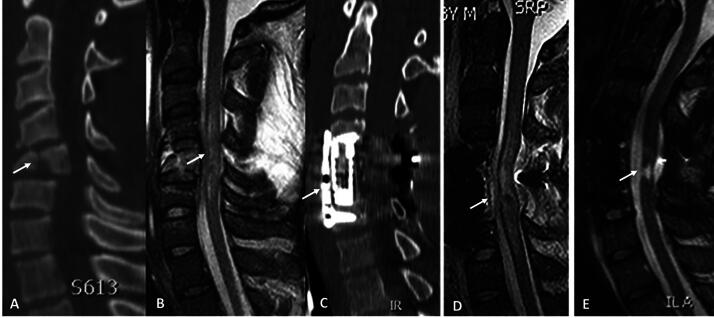
Index case. Midsagittal CT cuts indicating C5 tear-drop fracture (arrow) with translation of C5 body into spinal canal **(A)**. Midsagittal T2-weighted MRI indicating reduction with hematomyelia and spinal cord swelling (arrow). Intramedullary lesion length (IMLL) was 49.7 mm **(B)**. C5 corpectomy and circumferential C4-C6 fusion was conducted (arrow) **(C)**. Immediate post-operative midsagittal T2-weighted MRI indicating spinal cord decompression (arrow) and IMLL of 62.7 mm **(D)**. Midsagittal T2-weighted MRI 6 months following trauma indicating a small syrinx (arrow) at C5 level **(E)**.

**FIG. 2.**
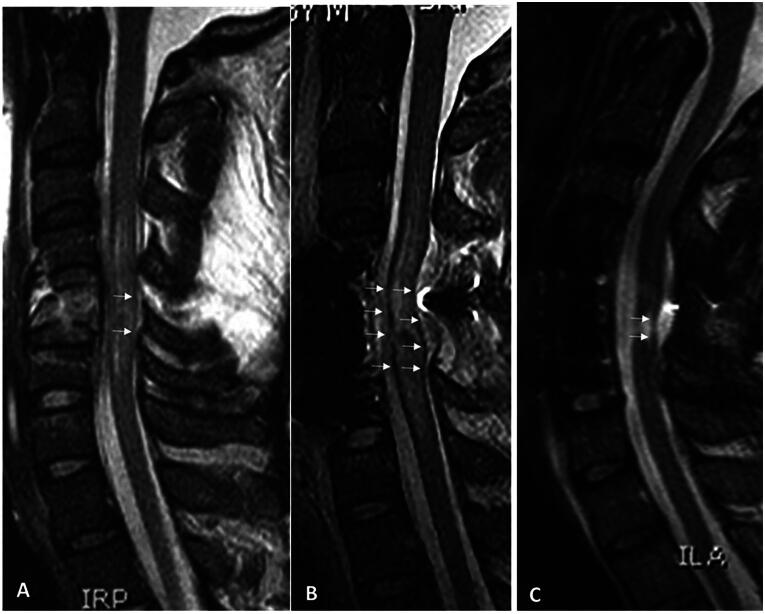
Index case tissue bridges. Midsagittal T2-weighted MRI cut indicating faint tissue bridges (arrows) in preoperative MRI **(A)**. Clear preservation of tissue bridges (arrows) on immediate post-operative T2-weighted MRI **(B)**. Maintained tissue bridges (arrows) 6 months following trauma in T2-weighted MRI **(C)** in conjunction with central syrinx.

*Index case*: A 56-year-old man suffered a recreational sport traumatic SCI and was rendered quadriplegic. He was airlifted to the R Adams Cowley Shock Trauma Center and was seen within 45 min in the Trauma Resuscitation Unit. Post-resuscitation ISNCSI exam revealed an ASIA motor score of 15 and ASIA Impairment grade of A. CT scan of the cervical spine obtained 2 h after trauma revealed a C5 tear-drop fracture (AO Class C; Fig. 1A) and T2-weighted MRI 6 h after trauma showed hematomyelia and spinal cord swelling (Fig. 1B). One-stage C5 corpectomy and C4 + C5 laminectomy and fusion (Fig. 1C) was performed. Post-operative MRI indicated spinal cord decompression and spinal cord swelling (Fig. 1D). MRI obtained nearly 6 months after injury indicated a small syrinx at the C5 level (Fig. 1E). Closer examination of MRIs indicates clear tissue bridges traversing the injury pre-operatively (Fig. 2A) that were preserved immediately post-operatively (Fig. 2B) and at 6-month follow-up (Fig. 2C). At 6-month follow-up, the patient’s ASIA motor score improved to 56 and AIS grade C.

Careless use of nomenclature may have resulted in the index patient being erroneously labeled as harboring a “complete SCI,” resulting in an overly grim prognosis delivered to the patient and his family. Instead, due to the phenomenon of neuronal stunning of axons in the preserved tissue bridges, this patient harbored a functionally complete yet anatomically incomplete injury. Assisted by proper medical and surgical management, he later underwent AIS grade conversion. Careful use of nomenclature is essential to avoid unwarranted clinical nihilism.

### Basic concepts of secondary injury

Secondary injury is widely recognized to be an important contributor to tissue loss after SCI. The concept of secondary injury originated in 1911 from experiments conducted by Major Alfred Reginald Allen, who developed the first experimental model of SCI. Dr. Allen noticed that evacuation of the contused hemorrhagic tissue led to functional improvement and postulated that some “noxious agent” was released by the injured tissue that led to autodestruction of surrounding neural tissue.^91^ Tragically, he died at the age of 42 from a shrapnel injury during the Meuse-Argonne Offensive of World War I. Later ultrastructural studies showed that, following experimental spinal cord contusion, initially intact tissue surrounding the lesion undergoes progressive changes including loss of endothelial integrity, erythrocyte extravasation, axonal swelling and degeneration, edema formation, and cystic degeneration.^18,19,92,93^

Despite its long history, the term secondary injury remains nebulously defined. Phenomena including ischemia, progression of hemorrhage, oxidative stress, and neuroinflammation have all been referred to as secondary injury in the literature. However, these phenomena represent causes or mechanisms of secondary injury rather than secondary injury itself. We define secondary injury as the delayed loss of functional neural tissue, including axons or neuronal somata, and the delayed loss of supportive cells such as glia or vasculature, which can contribute to neuronal loss. The time course of secondary injury after SCI varies depending on the mechanism. For example, in animal models, axonal swelling and loss is a relatively early event, with spheroid formation occurring within 1 h of injury, 70% of axonal loss occurring within the first day after injury, and continued loss throughout 2 weeks.^94^ In contrast, apoptotic cells are most frequently observed at ∼1 week after injury, although apoptosis is detectable as early as 6 h after injury.^95^

### AIS grade conversion is an integration of neuronal stunning and secondary injury

**FIG. 3.**
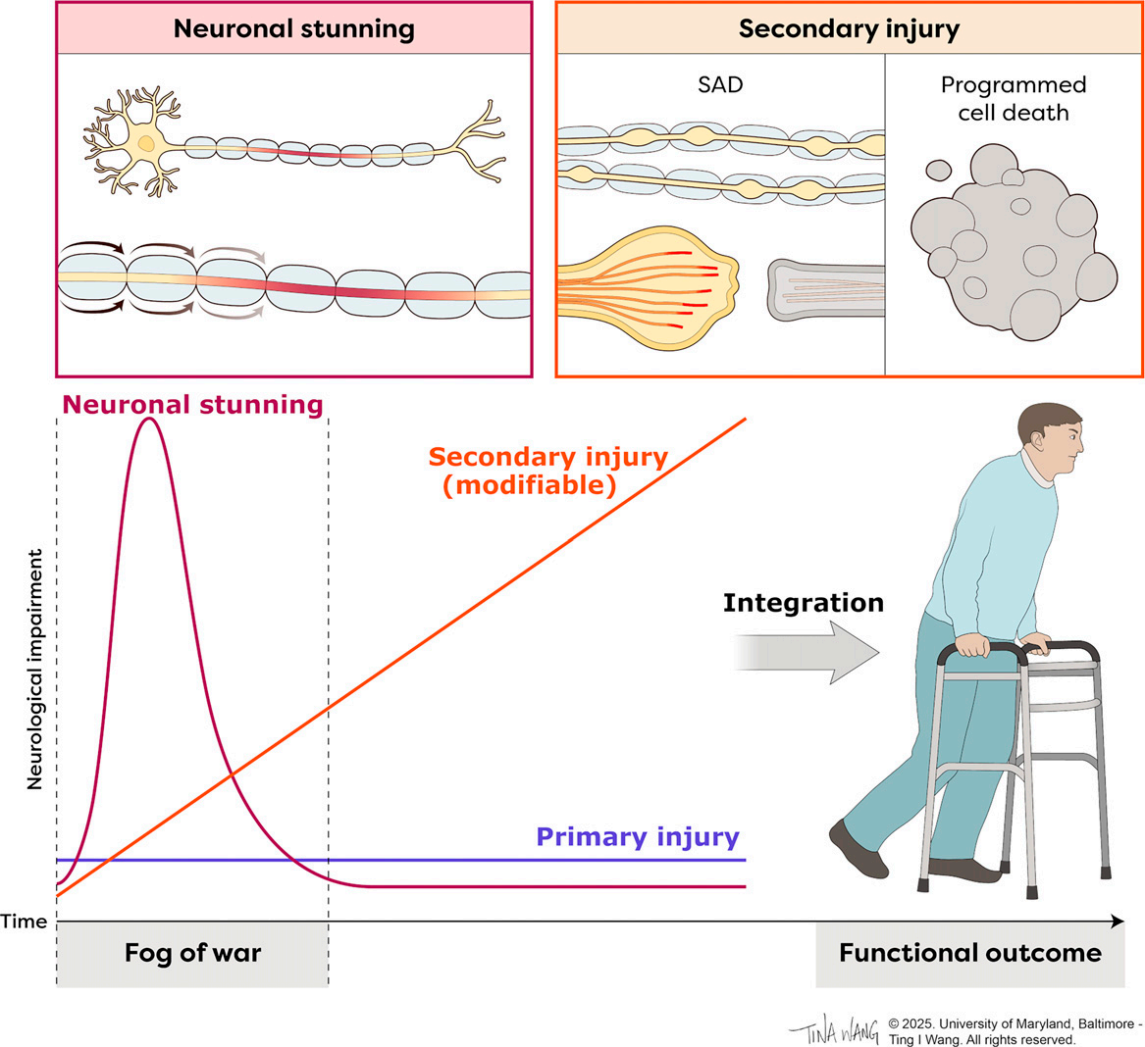
Neurological function is an integration of neuronal stunning, primary injury, and secondary injury. Illustration showing that neurological impairment at each time point and the neurological outcome after spinal cord injury reflects the integrated effects of primary injury, secondary injury, and neuronal stunning. Neuronal stunning, which arises from conduction block and hyperpolarization, is a reversible phenomenon. Secondary injury due to SAD and programmed cell death is potentially preventable yet irreversible once it occurs and primary injury is neither preventable nor reversible. Neuronal stunning may obscure the extent of primary injury and ongoing secondary injury, i.e., the “fog of war,” resulting in neurological examinations that do not fully predict potential functional recovery. SAD, secondary axonal degeneration.

Secondary injury reduces probably the upward AIS grade conversion by progressively reducing the underlying population of potentially viable neural tissue. Since a mere 5–10% salvage of white matter volume can translate to substantial improvement in functional outcome,^96,97^ the aggressive prevention of tissue losses from secondary injury is of the highest priority. Secondary injury may co-associate with neuronal stunning, which may both mimic and potentially “hide” ongoing secondary injury, so that it is not always possible at the bedside to distinguish secondary injury from transient neuronal stunning. Only after the “fog of war” of neuronal stunning has subsided can the true extent of primary and secondary injury be fully appreciated, although it remains difficult to determine the amount of potentially salvageable neural tissue that is lost (see above). The integration of these phenomena therefore manifests as AIS grade conversion (Fig. 3). As a consequence of the entanglement of secondary injury and neuronal stunning, neurological deficits cannot be simply ascribed to stunning but rather should be aggressively treated as if they may become permanent without medical intervention. In the following sections, key mechanisms of secondary injury are individually described.

### Programmed cell death contributes to secondary injury

Multiple mechanisms of cellular death contribute to neuronal loss after SCI. While reviews exist that describe these mechanisms in detail,^98,99^ here, we briefly discuss their relevance to secondary injury after SCI. Of note, the multifactorial nature and variety of mechanisms of cell death after SCI may explain the lack of success of specific inhibitors.

Apoptosis, a form of programmed cell death that involves membrane blebbing, nuclear fragmentation, cell shrinkage, and plasmalemmal exposure of phosphatidylserine, may be triggered by intrinsic or extrinsic factors.^98^ During intrinsic (mitochondrial) apoptosis, intracellular stress precipitates permeabilization of the outer mitochondrial membrane, cytochrome C release, and activation of the apoptosome and caspase-9, -7, and -3, with subsequent execution.^98^ Extrinsic apoptosis is triggered by signaling death ligands including fas ligand or tumor necrosis factor (TNF), which activates caspase-8 and subsequently activates caspases-7 and -3, with execution.^98^ Importantly, apoptosis does not include cell lysis and is therefore less inflammatory than other forms of programmed cell death. Apoptosis is widely detected in multiple different cell types after experimental and human SCI.^100^ Neuronal apoptosis peaks at early time points within the first day after injury, whereas a second wave of apoptosis occurs in glial white matter cells several days after injury and is associated with white matter tract degeneration.^101–103^

Pyroptosis is a form of inflammatory cell death originally characterized in bacterial infections. In pyroptosis, extracellular markers of infection or disease, such as pathogen- or damage-associated molecular patterns (DAMPs), engage cell surface receptors, resulting in activation of large complexes called inflammasomes. These inflammasomes activate caspase-1, resulting in plasmalemmal pore formation, cell swelling, and membrane rupture.^98^ After SCI, markers of pyroptosis are upregulated,^104,105^ and pyroptosis has been shown to contribute to post-SCI neuronal death.^106^

Necroptosis is a caspase-independent form of programmed cell death that is triggered classically by TNFα signaling and inflammation. Necroptosis exhibits many features similar to necrosis, with early membrane permeabilization and cellular and organelle swelling, but unlike necrosis relies on an active signaling cascade mechanism.^107,108^ Necroptosis has been shown to contribute to neuronal and glial cell death in SCI and worsens functional outcome.^109–111^

### Secondary axotomy contributes to secondary injury

Axon disconnection after injury can be caused by primary axotomy, which refers to the direct mechanical rending of axons that occurs at the time of injury, or secondary axotomy, a delayed form of axotomy that occurs as a consequence of SAD. Primary axotomy is not amenable to therapeutic intervention since the disconnection occurs at the time of injury (an ultrastructural analog of anatomical completeness). SAD is morphologically evident by the formation of periodic swellings or spheroids along the axons involved. Unlike primary axotomy, since SAD remains reversible in the initial stages and occurs in the hours after injury, it may be amenable to therapeutic intervention.^112–114^

SAD was originally described in traumatic brain injury (TBI). While axon retraction bulbs and axon swellings were observed in human specimens as early as 1900, they were presumed to be caused by direct mechanical rending of axons.^115^ In 1956, Sabina Strich described five patients with diffuse degeneration of the white matter and poor neurological outcome following TBI, despite a lack of intracranial hematoma or raised ICP.^116^ This led Thomas Gennarelli and others in the 1980s to describe and coin the term diffuse axonal injury (DAI).^117^ However, even at this time, axon degeneration after DAI was still presumed to be caused by direct mechanical rending of axons.^118^ It is only since the late 1980s and 1990s when John Povlishock and others showed that many axons remain initially in continuity after TBI but later undergo disconnection and that the role of SAD was appreciated.^119^ Primary axotomy is now believed to only occur after the most severe TBIs, whereas secondary axotomy likely represents the dominant cause of axon disconnection.

**FIG. 4.**
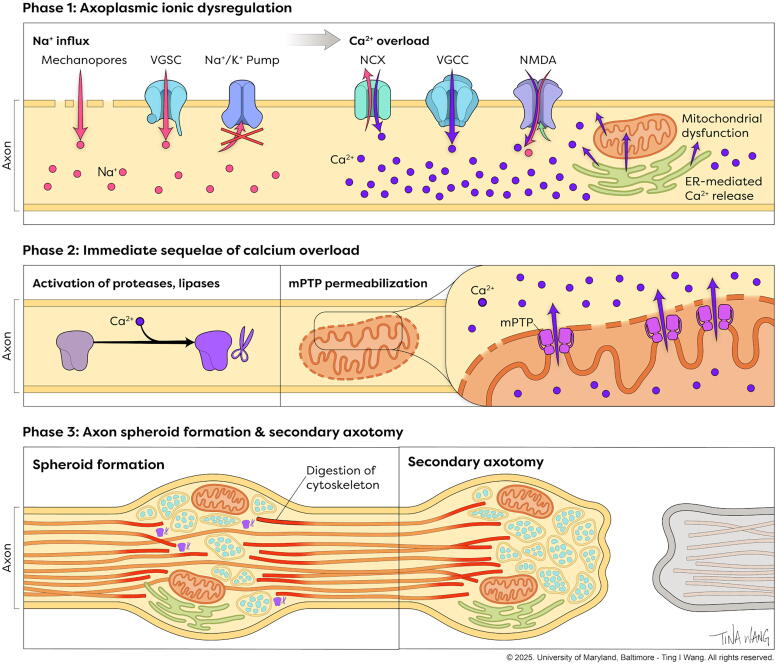
Phases of SAD. Illustration showing the phases of SAD. In phase 1, axoplasmic ionic dysregulation occurs with maladaptive overload of axoplasmic sodium and calcium. In phase 2, axoplasmic calcium overload triggers activation of calcium-sensitive proteases and lipases and mitochondrial permeability transition pore (mPTP) permeabilization, which further contributes to calcium overload and energy failure. In phase 3, protease-mediated degradation of the axon cytoskeleton results in spheroid formation and eventually in secondary axotomy. Prior to axotomy, this process is potentially reversible. SAD, secondary axonal degeneration.

Secondary axotomy and SAD also occur in SCI,^94^ where axons undergoing SAD exhibit similar morphological changes to those seen after TBI, including formation of axon swellings and endbulbs.^94,113^ As in TBI, the axons undergoing SAD after SCI are potentially salvageable in the early stages, with one study showing spontaneous resolution of ∼40% of spheroids after injury.^94^ Given the importance of white matter to functional outcomes after SCI,^96,97^ SAD is an important target for therapeutic intervention. Of note, SAD and secondary axotomy may be particularly relevant in tissue bridges in that these processes would shrink the pool of potentially recoverable neural elements. Since SAD and secondary axotomy are also triggered by hypoxia, timely surgical decompression and avoidance of systemic hypotension may improve outcomes in part through axonoprotection.^120^ While the detailed molecular mechanisms currently remain unclear, SAD appears to occur in three major phases discussed below, which may be part of a general axonal response to mechanical injury (Fig. 4).

#### Phase 1: Axoplasm ionic dysregulation

The first phase of SAD is the dysregulation of axoplasm ion homeostasis. Within minutes of injury, axoplasmic sodium quickly elevates.^121^ There are multiple potential routes for sodium influx. The so-called mechanoporation of the axolemma, where temporary pores form in the normally impermeable plasma membrane, may allow for translocation of ions and larger molecules such as charged proteins such as horseradish peroxidase.^122–124^ Notably, mechanoporation remains controversial, with some authors arguing that these observations may simply arise from the severed and open ends of primarily axotomized axons.^125,126^ Sodium overload also arises from inhibition of the Na^+^/K^+^/ATPase secondary to energy failure and transmembrane influx through VGSC.^127,128^ VGSCs are mechanosensitive,^129,130^ and when axonal stretch is applied, these channels convert from slow to fast current kinetics. Pharmacological inhibition of VGSCs following axonal injury has been shown to reduce sodium and calcium influx.^121,125,131^ Interestingly, activation of VGSCs is augmented after injury due to calcium-mediated proteolysis of an intracellular loop, resulting in the loss of inactivation.^132–134^

Sodium influx precedes and causes calcium influx,^131,132^ which is a key event after axonal injury. Axoplasmic calcium overload occurs through influx across the axolemma and also through intracellular store release.^135^ Axoplasmic calcium overload is synchronous with a decrease of extracellular calcium, highlighting the importance of extracellular sources.^121,125,136,137^ This occurs through multiple plasmalemmal channels and transporters. NCX exchangers typically function in the so-called forward mode where calcium is exported and sodium imported. Normally, NCX1 is localized to perinodal regions where it contributes to calcium buffering.^138^ After injury, NCX1 contributes to axon calcium overload^125,133,139,140^ and SAD.^141^ During intracellular sodium overload, NCX exchanges operate in reverse mode, where sodium efflux becomes linked to calcium influx. Multiple subtypes of voltage gated calcium channel (VGCC) and N-methyl-D-aspartate (NMDA) receptors are also implicated in neuron degeneration and calcium influx after axon injury.^125,142–144^ Intracellular release of calcium stores is also a major contributor to calcium overload. Calcium release from the axoplasmic reticulum contributes to calcium overload after injury, and inhibition of axoplasmic reticulum calcium release is protective in models of SCI.^145–150^ Axonal mitochondria also release calcium after injury, further contributing to overload.^145^

#### Phase 2: Immediate sequelae of calcium overload

Axoplasmic calcium overload is a key pathological event in SAD. After SCI, dysregulation of intracellular calcium is independently predictive of axonal loss, and depletion of extracellular calcium is axonoprotective.^113^ Calcium overload triggers an array of sequelae that contribute to axon degeneration, including activation of calcium-sensitive proteases and lipases, including calpains, caspases, phospholipase A2, lipoxygenase, and cyclooxygenase.^151,152^ Mitochondrial dysregulation and protease activation are two immediate sequelae of calcium overload that are particularly pertinent to axon injury and represent potential targets of therapeutic intervention.

Calcium overload leads to mitochondria dysregulation, which contributes to axonal degeneration. Axoplasmic calcium overload triggers permeabilization of the mPTP, which results in loss of the mitochondrial membrane potential, energetic failure, and additional release of intracellular calcium.^153,154^ Inhibition of mitochondria permeability transition pore (mPTP) protects axons from degeneration and contributes to axon degeneration through release of cytochrome-c and activation of caspases and calpains.^155,156^

Axoplasmic calcium overload also triggers calpain protease activation. Calpains are a family of calcium-activated intracellular proteases that are activated after axon injury and contribute to SAD.^157^ The most common calpain isoforms are µ- and m-calpain, which are activated by micromolar and millimolar levels of calcium, respectively.^157^ All neurofilament isoforms, microtubules, and spectrin are calpain substrates, and calpain-mediated cytoskeletal breakdown represents a key event in SAD.^157^ After axonal injury, calcium influx is spatially correlated with calpain activation, which is detected in axons as early as 90 min after injury and is present up to 1 week after injury.^124,158,159^ Blockade of sodium and calcium influx protects against calpain activation.^144^ Calpain activation is correlated with cytoskeletal degradation and neurofilament compaction, and calpain blockade protects against axonal degeneration.^113,160–165^ Importantly, pan-calpain inhibition may not be therapeutic, as some calpain isoforms yield neuroprotective effects.^166^

#### Phase 3: Axon spheroid formation and secondary axotomy

As SAD progresses, a subset of injured axons demonstrate morphological changes, wherein focal regions of axon swelling form, a phenomenon also referred to as axon spheroid formation. Spheroid formation was first described in 1983 in a cat model of mild TBI^167^ and later confirmed in human specimens.^168^ In these tissues, direct mechanical disconnection of axons, microhemorrhage, and parenchymal disruption is often absent.^119,167,169^

Axon swellings primarily occur at internodal regions and reflect an abnormal local accumulation of organelles, ultimately caused by a local breakdown of anterograde transport due to cytoskeletal disruption.^170,171^ Calpain-mediated cytoskeletal breakdown plays a major role in this process.^124^ Nodal regions also exhibit other early morphological changes, including formation of nodal blebs and loss of subaxolemmal density.^172^

Starting at 3–6 h and peaking at 12–24 h after injury, a subset of axon swellings exhibit constrictions and multilobulations.^167,169,172^ These constrictions deepen, eventually dividing the axon and completing the process of SAD with secondary axotomy, with subsequent degradation of the distal part of the axon. Interestingly, while secondary axotomy is most frequent during the hours-to-days after injury, axonal swellings are also detected at more chronic time points after injury, indicating ongoing delayed degeneration.^173,174^ While the majority of studies have been conducted in human TBI and TBI animal models, similar changes occur after SCI and peripheral nerve injury,^94,175^ indicating that SAD is a general form of delayed axonal degeneration.

Immunohistochemical markers have been developed to detect secondary axotomy in postmortem tissues. β amyloid precursor protein (β-APP), a widely expressed anterograde transported axonal protein, is currently the most sensitive and best validated marker for SAD.^176–178^ β-APP immunolabeling accumulates at regions of axonal swellings and disconnection due to local breakdown of anterograde transport and is a useful marker for the aforementioned morphological changes.^170,179^ There are several important limitations for β-APP immunolabeling that should be considered. For unclear reasons, β-APP immunolabeling only identifies a subset of traumatically injured axons,^180^ is lost after a few days in the proximal axon,^181^ is not present in the distal axon,^169^ and may therefore underrepresent total axonal damage in some cases.^182^

SAD and spheroid formation is reversible during the early stages prior to axon disconnection.^112–114,183^ Axons that exhibit focal swellings are vulnerable but remain potentially viable. In one study of SAD following experimental SCI, more than 40% of axons exhibiting focal swellings exhibited spontaneous recovery during the course of the experiment.^94^ While SAD contributes to secondary injury by driving secondary axotomy, it also contributes to neuronal stunning in that axons undergoing early stages of SAD suffer from conduction block. Since up to 40% of axons exhibiting spheroid formation can recover, this conduction block may be resolved, manifesting as AIS grade conversion.

### Factors that worsen or ameliorate secondary injury

Programmed cell death and secondary axotomy represent the core underlying mechanisms of secondary injury. Associated pathophysiological processes including ischemia, inflammation, edema, and hemorrhage exacerbate these processes by creating a hostile extracellular milieu in which the chance of axon and cell survival is diminished. Many of these factors are also potentially modifiable with clinical interventions.

Tissue ischemia and hypoperfusion are major drivers of secondary injury after SCI. Following SCI, tissue perfusion becomes progressively impaired in both grey and white matter.^184–188^ In axons and neurons, ischemia results in increased intracellular sodium and loss of intracellular potassium consistent with dysregulation of the Na/K/ATPase.^127,128^ This culminates in increased intracellular calcium and degeneration, which further worsens programmed cell death and secondary axotomy as described above.^127,128^ Ischemia and hypoxia therefore greatly compound post-traumatic neuronal and axonal loss.^189^ Early and complete surgical decompression and avoidance of hypotension after SCI are the current mainstays of treatment to mitigate the negative effects of ischemia and hypoperfusion and promote survival of neural tissue. There is ongoing debate regarding the precise timing and method of surgical decompression. The STASCIS trial showed that early (within 24 h from injury) surgical decompression improved the odds of AIS grade conversion in patients with cervical SCI,^74^ a finding that has been reproduced in subsequent studies.^190–192^ It remains unclear whether additional benefits might be reaped from ultra-early surgery, in which surgery is pursued at earlier timepoints after injury.^191^ With regard to surgical technique for decompression, posterior laminectomy yields increased decompression rates versus anterior fixation.^193^

Under physiological conditions, the spinal cord is an immune privileged organ. However, injury precipitates a local release of DAMPs, which stimulate the innate immune system. This triggers a wave of neutrophils and later monocytes and macrophages to invade the damaged spinal cord.^194^ At later timepoints, the adaptive immune system also contributes to neuroinflammation by the presence of NK-, B-, and T-cells. For reasons that are not entirely clear, SCI generates a relatively greater magnitude of immune response compared with TBI.^195–197^ Although neuroinflammation contributes to debris clearance and is not purely maladaptive, many immune cells also have cytotoxic effects. Neutrophil degranulation releases myeloperoxidase, which generate free radicals within the contusion, contributing to glial and neuronal apoptosis beyond the injury epicenter.^198^ Neutrophil extracellular traps composed of granule proteins and chromatin are expelled from the neutrophils and cause additional apoptosis and fibrosis.^199,200^ Classically activated monocytes and macrophages also contribute to secondary injury by secreting neurotoxic compounds including interleukin (IL)-1, TNF-α, and IL-6, which further propagate inflammation, contribute to blood–spinal cord barrier breakdown, and stimulate astrogliosis and glial scarring.^201–204^ These monocytes and macrophages also secrete nicotinamide adenine dinucleotide phosphate (NADPH) oxidase (NOX) and nitric oxide synthase (NOS2), which lead to oxidative stress.^203,205,206^ Axon dieback, the phenomenon in which axonal growth cones undergo rapid retraction, has been connected to the presence of pro-inflammatory macrophages in the injured spinal cord.^207,208^ While the national acute spinal cord injury study (NASCIS) trials have studied methylprednisolone to reduce neuroinflammation in the NASCIS trials,^209,210^ controversy over potential lack of efficacy and side effects have prevented widespread adoption.

Contusion expansion and edema formation are key aspects that promote secondary injury in SCI. Contusive lesions appear within minutes after SCI and progressively expand due to progressive hemorrhagic necrosis.^18,19,211^ In the hours after injury, small petechial hemorrhages form in the normal parenchyma surrounding an existing contusion. These petechial hemorrhages may grow in size and merge, thereby expanding the front of the original contusion. Mechanistically, maladaptive *de novo* upregulation of the SUR1-TRPM4 channel by peri-lesional capillaries mediates oncotic endothelial death and capillary rupture due to influx of water.^19,212^ Edema accumulates in the spinal cord within minutes of injury and persists for 20 or more days.^213,214^ The extent of edema is proportional to injury severity.^215^ Mechanisms driving edema formation are diverse and reviewed elsewhere.^216,217^ The presence and expansion of progressive hemorrhagic necrosis and edema can be observed radiographically in human SCI as a region of T2 hyperintensity centered at the lesion epicenter. This area of T2 signal is usually measured and summarized by the intramedullary lesion length (IMLL) or BASIC score. In human SCI, IMLL expands at a rate of approximately 1000 µm/h in patients with AIS grades A and B injuries and 21 µm/h in AIS grades C and D injuries.^218,219^ Edema and hemorrhage together worsen the odds of a positive AIS grade conversion. IMLL expansion is strongly associated with AIS grade conversion, with a 10-mm increase in IMLL being linked with a 40% decreased odds of AIS grade conversion.^58^ Blood itself contains multiple directly neurotoxic components that are active at physiological concentrations.^220^ As a consequence, spinal cord hemorrhage directly results in axon and neuronal death.^221^ Both edema and hemorrhage contribute to increased intrathecal pressure and can worsen tissue perfusion, indirectly contributing to secondary injury.^214^

## Conclusions

For patients with SCI, the possibility of undergoing AIS grade conversion represents a flame of hope during what is likely the worst period of their lives. AIS grade conversion is an integration of the effects of primary injury, secondary injury, and neurological stunning and does not represent “healing” of neurological tissues. The key modifiable factor contributing to AIS grade conversion is secondary injury, which is comprised of programmed cell death of neuronal somata and SAD of axons. Currently, the only interventions to decrease secondary injury are timely and complete surgical decompression and avoidance of hypotension. These interventions are mechanistically nonspecific and have remained unchanged for decades. While we have developed some insight into the mechanisms of programmed cell death and SAD after SCI, more work is needed before targeted therapies can be developed and translated into widespread clinical use.

## Transparency, Rigor, and Reproducibility Summary

Since this article is a review article, it was not preregistered. No animals were used for this study. No statistical testing, blinding, or power analysis was required for this study. Further information is available on request of the corresponding author.

## Abbreviations Used

AIS: ASIA Impairment Scale
β-APP: β amyloid precursor protein
CNS: Central nervous system
DAMPs: Damage-associated molecular patterns
DAI: Diffuse axonal injury
IMLL: Intramedullary lesion length
mPTP: Mitochondria permeability transition pore
NADPH: Nicotinamide adenine dinucleotide phosphate
NASCIS: National acute spinal cord injury study
NCX: Sodium-calcium exchanger
NMDA: N-methyl-D-aspartate
NLI: Neurological level of injury
NOS: Nitric oxide synthase
NOX: NADPH oxidase
SAD: Secondary axonal degeneration
SER: Smooth endoplasmic reticulum
SCI: Spinal cord injury
SUR1-TRPM4: Sulfonylurea receptor 1–transient receptor potential melastatin 4
TBI: Traumatic brain injury
TNF: Tumor necrosis factor
VGCC: Voltage gated calcium channels
VGSC: Voltage gated sodium channels

## References

[1] Barbiellini Amidei C, Salmaso L, Bellio S, et al. Epidemiology of traumatic spinal cord injury: A large population-based study. Spinal Cord 2022;60(9):812–819; doi: 10.1038/s41393-022-00795-w 35396455 PMC8990493

[2] Ding W, Hu S, Wang P, et al. Spinal cord injury: The global incidence, prevalence, and disability from the global burden of disease study 2019. Spine (Phila Pa 1976) 2022;47(21):1532–1540; doi: 10.1097/BRS.0000000000004417 35857624 PMC9554757

[3] Lu Y, Shang Z, Zhang W, et al. Global incidence and characteristics of spinal cord injury since 2000-2021: A systematic review and meta-analysis. BMC Med 2024;22(1):285; doi: 10.1186/s12916-024-03514-9 38972971 PMC11229207

[4] Aarabi B, Albrecht JS, Simard JM, et al. Trends in demographics and markers of injury severity in traumatic cervical spinal cord injury. J Neurotrauma 2021;38(6):756–764; doi: 10.1089/neu.2020.7415 33353454

[5] Chen Y, He Y, DeVivo MJ. Changing demographics and injury profile of new traumatic spinal cord injuries in the United States, 1972-2014. Arch Phys Med Rehabil 2016;97(10):1610–1619; doi: 10.1016/j.apmr.2016.03.017 27109331

[6] McCaughey EJ, Purcell M, McLean AN, et al. Changing demographics of spinal cord injury over a 20-year period: A longitudinal population-based study in Scotland. Spinal Cord 2016;54(4):270–276; doi: 10.1038/sc.2015.167 26458974 PMC5399148

[7] Liu Y, Yang X, He Z, et al. Spinal cord injury: Global burden from 1990 to 2019 and projections up to 2030 using Bayesian age-period-cohort analysis. Front Neurol 2023;14:1304153; doi: 10.3389/fneur.2023.1304153 38116113 PMC10729761

[8] Bennett J, Das JM, Emmady PD. Spinal Cord Injuries. In: StatPearls. StatPearls: Treasure Island (FL) ineligible companies. Disclosure: Joe Das declares no relevant financial relationships with ineligible companies. Disclosure: Prabhu Emmady declares no relevant financial relationships with ineligible companies; 2024.

[9] Malekzadeh H, Golpayegani M, Ghodsi Z, et al. Direct cost of illness for spinal cord injury: A systematic review. Global Spine J 2022;12(6):1267–1281; doi: 10.1177/21925682211031190 34289308 PMC9210246

[10] Cao Y, Krause JS. Estimation of indirect costs based on employment and earnings changes after spinal cord injury: An observational study. Spinal Cord 2020;58(8):908–913; doi: 10.1038/s41393-020-0447-1 32139887

[11] Fehlings MG, Pedro K, Hejrati N. Management of acute spinal cord injury: Where have we been? Where are we now? Where are we going? J Neurotrauma 2022;39(23–24):1591–1602; doi: 10.1089/neu.2022.0009 35686453

[12] Chryssikos T, Stokum JA, Ahmed AK, et al. Surgical decompression of traumatic cervical spinal cord injury: A pilot study comparing real-time intraoperative ultrasound after laminectomy with postoperative MRI and CT Myelography. Neurosurgery 2023;92(2):353–362; doi: 10.1227/neu.0000000000002207 36637270 PMC9815093

[13] Schumacher PA, Siman RG, Fehlings MG. Pretreatment with calpain inhibitor CEP-4143 inhibits calpain I activation and cytoskeletal degradation, improves neurological function, and enhances axonal survival after traumatic spinal cord injury. J Neurochem 2000;74(4):1646–1655; doi: 10.1046/j.1471-4159.2000.0741646.x 10737623

[14] Tomes DJ, Agrawal SK. Role of Na(+)-Ca(2+) exchanger after traumatic or hypoxic/ischemic injury to spinal cord white matter. Spine J 2002;2(1):35–40; doi: 10.1016/s1529-9430(01)00151-6 14588286

[15] Teng YD, Wrathall JR. Local blockade of sodium channels by tetrodotoxin ameliorates tissue loss and long-term functional deficits resulting from experimental spinal cord injury. J Neurosci 1997;17(11):4359–4366; doi: 10.1523/JNEUROSCI.17-11-04359.1997 9151752 PMC6573566

[16] Stutzmann JM, Pratt J, Boraud T, et al. The effect of riluzole on post-traumatic spinal cord injury in the rat. Neuroreport 1996;7(2):387–392; doi: 10.1097/00001756-199601310-00003 8730788

[17] Rosenberg LJ, Teng YD, Wrathall JR. Effects of the sodium channel blocker tetrodotoxin on acute white matter pathology after experimental contusive spinal cord injury. J Neurosci 1999;19(14):6122–6133; doi: 10.1523/JNEUROSCI.19-14-06122.1999 10407048 PMC6783088

[18] Simard JM, Woo SK, Norenberg MD, et al. Brief suppression of Abcc8 prevents autodestruction of spinal cord after trauma. Sci Transl Med 2010;2(28):28ra29; doi: 10.1126/scitranslmed.3000522 PMC2903041 20410530

[19] Simard JM, Tsymbalyuk O, Ivanov A, et al. Endothelial sulfonylurea receptor 1-regulated NC Ca-ATP channels mediate progressive hemorrhagic necrosis following spinal cord injury. J Clin Invest 2007;117(8):2105–2113; doi: 10.1172/JCI32041 17657312 PMC1924498

[20] Zipser CM, Cragg JJ, Guest JD, et al. Cell-based and stem-cell-based treatments for spinal cord injury: Evidence from clinical trials. Lancet Neurol 2022;21(7):659–670; doi: 10.1016/S1474-4422(21)00464-6 35569486

[21] Burns AS, Marino RJ, Kalsi-Ryan S, et al. Type and timing of rehabilitation following acute and subacute spinal cord injury: A systematic review. Global Spine J 2017;7(3 Suppl):175S–194S; doi: 10.1177/2192568217703084 29164023 PMC5684843

[22] Levett JJ, Elkaim LM, Niazi F, et al. Invasive brain computer interface for motor restoration in spinal cord injury: A systematic review. Neuromodulation 2024;27(4):597–603; doi: 10.1016/j.neurom.2023.10.006 37943244

[23] Henmar S, Simonsen EB, Berg RW. What are the gray and white matter volumes of the human spinal cord? J Neurophysiol 2020;124(6):1792–1797; doi: 10.1152/jn.00413.2020 33085549

[24] Harrow-Mortelliti M, Reddy V, Jimsheleishvili G. Physiology, Spinal Cord. In: StatPearls. StatPearls: Treasure Island (FL) relationships with ineligible companies. Disclosure: Vamsi Reddy declares no relevant financial relationships with ineligible companies. Disclosure: George Jimsheleishvili declares no relevant financial relationships with ineligible companies; 2024. 31334987

[25] Hochman S. Spinal cord. Curr Biol 2007;17(22):R950–R5; doi: 10.1016/j.cub.2007.10.014 18029245

[26] Kaiser JT, Lugo-Pico JG. Neuroanatomy, Spinal Nerves. In: StatPearls. StatPearls: Treasure Island (FL) ineligible companies. Disclosure: Julian Lugo-Pico declares no relevant financial relationships with ineligible companies; 2024. 31194375

[27] Polilov AA. The smallest insects evolve anucleate neurons. Arthropod Struct Dev 2012;41(1):29–34; doi: 10.1016/j.asd.2011.09.001 22078364

[28] Perge JA, Koch K, Miller R, et al. How the optic nerve allocates space, energy capacity, and information. J Neurosci 2009;29(24):7917–7928; doi: 10.1523/JNEUROSCI.5200-08.2009 19535603 PMC2928227

[29] Prokop A. Cytoskeletal organization of axons in vertebrates and invertebrates. J Cell Biol 2020;219(7); doi: 10.1083/jcb.201912081 PMC7337489 32369543

[30] Smith G, Sweeney ST, O’Kane CJ, et al. How neurons maintain their axons long-term: An integrated view of axon biology and pathology. Front Neurosci 2023;17:1236815; doi: 10.3389/fnins.2023.1236815 37564364 PMC10410161

[31] Price RL, Paggi P, Lasek RJ, et al. Neurofilaments are spaced randomly in the radial dimension of axons. J Neurocytol 1988;17(1):55–62; doi: 10.1007/BF01735377 3418356

[32] Dubey S, Bhembre N, Bodas S, et al. The axonal actin-spectrin lattice acts as a tension buffering shock absorber. Elife 2020;9; doi: 10.7554/eLife.51772 PMC7190353 32267230

[33] Xu K, Zhong G, Zhuang X. Actin, spectrin, and associated proteins form a periodic cytoskeletal structure in axons. Science 2013;339(6118):452–456; doi: 10.1126/science.1232251 23239625 PMC3815867

[34] Leterrier C. Putting the axonal periodic scaffold in order. Curr Opin Neurobiol 2021;69:33–40; doi: 10.1016/j.conb.2020.12.015 33450534

[35] Misgeld T, Schwarz TL. Mitostasis in neurons: Maintaining mitochondria in an extended cellular architecture. Neuron 2017;96(3):651–666; doi: 10.1016/j.neuron.2017.09.055 29096078 PMC5687842

[36] Zhu XH, Qiao H, Du F, et al. Quantitative imaging of energy expenditure in human brain. Neuroimage 2012;60(4):2107–2117; doi: 10.1016/j.neuroimage.2012.02.013 22487547 PMC3325488

[37] Baughman JM, Perocchi F, Girgis HS, et al. Integrative genomics identifies MCU as an essential component of the mitochondrial calcium uniporter. Nature 2011;476(7360):341–345; doi: 10.1038/nature10234 21685886 PMC3486726

[38] De Stefani D, Raffaello A, Teardo E, et al. A forty-kilodalton protein of the inner membrane is the mitochondrial calcium uniporter. Nature 2011;476(7360):336–340; doi: 10.1038/nature10230 21685888 PMC4141877

[39] Choi S, Quan X, Bang S, et al. Mitochondrial calcium uniporter in Drosophila transfers calcium between the endoplasmic reticulum and mitochondria in oxidative stress-induced cell death. J Biol Chem 2017;292(35):14473–14485; doi: 10.1074/jbc.M116.765578 28726639 PMC5582840

[40] Tsukita S, Ishikawa H. Three-dimensional distribution of smooth endoplasmic reticulum in myelinated axons. J Electron Microsc (Tokyo) 1976;25(3):141–149. 1025229

[41] Luarte A, Cornejo VH, Bertin F, et al. The axonal endoplasmic reticulum: One organelle-many functions in development, maintenance, and plasticity. Dev Neurobiol 2018;78(3):181–208; doi: 10.1002/dneu.22560 29134778

[42] Raffaello A, Mammucari C, Gherardi G, et al. Calcium at the center of cell signaling: Interplay between endoplasmic reticulum, mitochondria, and lysosomes. Trends Biochem Sci 2016;41(12):1035–1049; doi: 10.1016/j.tibs.2016.09.001 27692849 PMC5123979

[43] Cho Y, Sloutsky R, Naegle KM, et al. Injury-induced HDAC5 nuclear export is essential for axon regeneration. Cell 2013;155(4):894–908; doi: 10.1016/j.cell.2013.10.004 24209626 PMC3987749

[44] Wenzel EM, Elfmark LA, Stenmark H, et al. ER as master regulator of membrane trafficking and organelle function. J Cell Biol 2022;221(10); doi: 10.1083/jcb.202205135 PMC9481738 36108241

[45] Pedraza L, Huang JK, Colman DR. Organizing principles of the axoglial apparatus. Neuron 2001;30(2):335–344; doi: 10.1016/s0896-6273(01)00306-3 11394997

[46] Rosenbluth J. Intramembranous particle distribution at the node of Ranvier and adjacent axolemma in myelinated axons of the frog brain. J Neurocytol 1976;5(6):731–745; doi: 10.1007/BF01181584 1087339

[47] Chiu SY, Ritchie JM. Evidence for the presence of potassium channels in the paranodal region of acutely demyelinated mammalian single nerve fibres. J Physiol 1981;313:415–437; doi: 10.1113/jphysiol.1981.sp013674 6268773 PMC1274460

[48] Maynard FM, Jr, Bracken MB, Creasey G, et al. International standards for neurological and functional classification of spinal cord injury. American Spinal Injury Association. Spinal Cord 1997;35(5):266–274; doi: 10.1038/sj.sc.3100432 9160449

[49] Furlan JC, Noonan V, Singh A, et al. Assessment of impairment in patients with acute traumatic spinal cord injury: A systematic review of the literature. J Neurotrauma 2011;28(8):1445–1477; doi: 10.1089/neu.2009.1152 20030559 PMC3143408

[50] Kirshblum S, Snider B, Eren F, et al. Characterizing natural recovery after traumatic spinal cord injury. J Neurotrauma 2021;38(9):1267–1284; doi: 10.1089/neu.2020.7473 33339474 PMC8080912

[51] Herbison GJ, Zerby SA, Cohen ME, et al. Motor power differences within the first two weeks post-SCI in cervical spinal cord-injured quadriplegic subjects. J Neurotrauma 1992;9(4):373–380; doi: 10.1089/neu.1992.9.373 1291696

[52] Brown PJ, Marino RJ, Herbison GJ, et al. The 72-hour examination as a predictor of recovery in motor complete quadriplegia. Arch Phys Med Rehabil 1991;72(8):546–548. 2059130

[53] Evaniew N, Sharifi B, Waheed Z, et al. The influence of neurological examination timing within hours after acute traumatic spinal cord injuries: An observational study. Spinal Cord 2020;58(2):247–254; doi: 10.1038/s41393-019-0359-0 31595042

[54] Blaustein DM, Zafonte R, Thomas D, et al. Predicting recovery of motor complete quadriplegic patients. 24 hour v 72 hour motor index scores. Am J Phys Med Rehabil 1993;72(5):306–311; doi: 10.1097/00002060-199310000-00010 8398023

[55] El Tecle NE, Dahdaleh NS, Bydon M, et al. The natural history of complete spinal cord injury: A pooled analysis of 1162 patients and a meta-analysis of modern data. J Neurosurg Spine 2018;28(4):436–443; doi: 10.3171/2017.7.SPINE17107 29350593

[56] Spiess MR, Muller RM, Rupp R, et al.; EM-SCI Study Group. Conversion in ASIA impairment scale during the first year after traumatic spinal cord injury. J Neurotrauma 2009;26(11):2027–2036; doi: 10.1089/neu.2008.0760 19456213

[57] Marino RJ, Burns S, Graves DE, et al. Upper- and lower-extremity motor recovery after traumatic cervical spinal cord injury: An update from the national spinal cord injury database. Arch Phys Med Rehabil 2011;92(3):369–375; doi: 10.1016/j.apmr.2010.09.027 21353821

[58] Aarabi B, Sansur CA, Ibrahimi DM, et al. Intramedullary lesion length on postoperative magnetic resonance imaging is a strong predictor of ASIA impairment scale grade conversion following decompressive surgery in cervical spinal cord injury. Neurosurgery 2017;80(4):610–620; doi: 10.1093/neuros/nyw053 28362913 PMC5748932

[59] Ditunno PL, Patrick M, Stineman M, et al. Who wants to walk? Preferences for recovery after SCI: A longitudinal and cross-sectional study. Spinal Cord 2008;46(7):500–506; doi: 10.1038/sj.sc.3102172 18209742

[60] Anderson KD. Targeting recovery: Priorities of the spinal cord-injured population. J Neurotrauma 2004;21(10):1371–1383; doi: 10.1089/neu.2004.21.1371 15672628

[61] van Middendorp JJ, Hosman AJ, Pouw MH, et al.; EM-SCI Study Group. ASIA impairment scale conversion in traumatic SCI: Is it related with the ability to walk? A descriptive comparison with functional ambulation outcome measures in 273 patients. Spinal Cord 2009;47(7):555–560; doi: 10.1038/sc.2008.162 19104512

[62] Ditunno JF, Scivoletto G, Patrick M, et al. Validation of the walking index for spinal cord injury in a US and European clinical population. Spinal Cord 2008;46(3):181–188; doi: 10.1038/sj.sc.3102071 17502878

[63] Scivoletto G, Tamburella F, Laurenza L, et al. Who is going to walk? A review of the factors influencing walking recovery after spinal cord injury. Front Hum Neurosci 2014;8:141; doi: 10.3389/fnhum.2014.00141 24659962 PMC3952432

[64] Steeves JD, Lammertse D, Curt A, et al.; International Campaign for Cures of Spinal Cord Injury Paralysis. Guidelines for the conduct of clinical trials for spinal cord injury (SCI) as developed by the ICCP panel: Clinical trial outcome measures. Spinal Cord 2007;45(3):206–221; doi: 10.1038/sj.sc.3102008 17179972

[65] Khan O, Badhiwala JH, Fehlings MG. Prediction of independence in bowel function after spinal cord injury: Validation of a logistic regression model. Spinal Cord 2021;59(2):207–214; doi: 10.1038/s41393-020-00551-y 32963361 PMC7870806

[66] Javeed S, Greenberg JK, Zhang JK, et al. Association of upper-limb neurological recovery with functional outcomes in high cervical spinal cord injury. J Neurosurg Spine 2023;39(3):355–362; doi: 10.3171/2023.4.SPINE2382 37243549

[67] Mautes AE, Weinzierl MR, Donovan F, et al. Vascular events after spinal cord injury: Contribution to secondary pathogenesis. Phys Ther 2000;80(7):673–687. 10869130

[68] Zariffa J, Kramer JL, Fawcett JW, et al. Characterization of neurological recovery following traumatic sensorimotor complete thoracic spinal cord injury. Spinal Cord 2011;49(3):463–471; doi: 10.1038/sc.2010.140 20938451

[69] Resnick DK. Updated guidelines for the management of acute cervical spine and spinal cord injury. Neurosurgery 2013;72(Suppl 2):1; doi: 10.1227/NEU.0b013e318276ee7e 23417171

[70] Hawryluk G, Whetstone W, Saigal R, et al. Mean arterial blood pressure correlates with neurological recovery after human spinal cord injury: Analysis of high frequency physiologic data. J Neurotrauma 2015;32(24):1958–1967; doi: 10.1089/neu.2014.3778 25669633 PMC4677564

[71] Walters BC, Hadley MN, Hurlbert RJ, et al.; Congress of Neurological Surgeons. Guidelines for the management of acute cervical spine and spinal cord injuries: 2013 update. Neurosurgery 2013;60(CN_suppl_1):82–91; doi: 10.1227/01.neu.0000430319.32247.7f 23839357

[72] Kwon BK, Tetreault LA, Martin AR, et al. A clinical practice guideline for the management of patients with acute spinal cord injury: Recommendations on hemodynamic management. Global Spine J 2024;14(3_suppl):187S–211S; doi: 10.1177/21925682231202348 38526923 PMC10964888

[73] Yousefifard M, Rahimi-Movaghar V, Baikpour M, et al. Early versus late spinal decompression surgery in treatment of traumatic spinal cord injuries; a systematic review and meta-analysis. Emerg (Tehran) 2017;5(1):e37. 28286844 PMC5325907

[74] Fehlings MG, Vaccaro A, Wilson JR, et al. Early versus delayed decompression for traumatic cervical spinal cord injury: Results of the Surgical Timing in Acute Spinal Cord Injury Study (STASCIS). PLoS One 2012;7(2):e32037; doi: 10.1371/journal.pone.0032037 22384132 PMC3285644

[75] Bourassa-Moreau E, Mac-Thiong JM, Li A, et al. Do patients with complete spinal cord injury benefit from early surgical decompression? Analysis of neurological improvement in a prospective cohort study. J Neurotrauma 2016;33(3):301–306; doi: 10.1089/neu.2015.3957 26494114

[76] Marino RJ, Leff M, Cardenas DD, et al. Trends in rates of ASIA impairment scale conversion in traumatic complete spinal cord injury. Neurotrauma Rep 2020;1(1):192–200; doi: 10.1089/neur.2020.0038 34223541 PMC8240895

[77] Raineteau O, Schwab ME. Plasticity of motor systems after incomplete spinal cord injury. Nat Rev Neurosci 2001;2(4):263–273; doi: 10.1038/35067570 11283749

[78] Zheng B, Tuszynski MH. Regulation of axonal regeneration after mammalian spinal cord injury. Nat Rev Mol Cell Biol 2023;24(6):396–413; doi: 10.1038/s41580-022-00562-y 36604586

[79] Onifer SM, Nunn CD, Decker JA, et al. Loss and spontaneous recovery of forelimb evoked potentials in both the adult rat cuneate nucleus and somatosensory cortex following contusive cervical spinal cord injury. Exp Neurol 2007;207(2):238–247; doi: 10.1016/j.expneurol.2007.06.012 17678895 PMC2141689

[80] James ND, Bartus K, Grist J, et al. Conduction failure following spinal cord injury: Functional and anatomical changes from acute to chronic stages. J Neurosci 2011;31(50):18543–18555; doi: 10.1523/JNEUROSCI.4306-11.2011 22171053 PMC3495307

[81] Nashmi R, Imamura H, Tator CH, et al. Serial recording of somatosensory and myoelectric motor evoked potentials: Role in assessing functional recovery after graded spinal cord injury in the rat. J Neurotrauma 1997;14(3):151–159; doi: 10.1089/neu.1997.14.151 9104932

[82] Marion CM, Radomski KL, Cramer NP, et al. Experimental traumatic brain injury identifies distinct early and late phase axonal conduction deficits of white matter pathophysiology, and reveals intervening recovery. J Neurosci 2018;38(41):8723–8736; doi: 10.1523/JNEUROSCI.0819-18.2018 30143572 PMC6181309

[83] Kolaric KV, Thomson G, Edgar JM, et al. Focal axonal swellings and associated ultrastructural changes attenuate conduction velocity in central nervous system axons: A computer modeling study. Physiol Rep 2013;1(3):e00059; doi: 10.1002/phy2.59 24303138 PMC3835014

[84] Redford EJ, Kapoor R, Smith KJ. Nitric oxide donors reversibly block axonal conduction: Demyelinated axons are especially susceptible. Brain 1997;120(Pt 12):2149–2157; doi: 10.1093/brain/120.12.2149 9448570

[85] Leis AA, Kronenberg MF, Stĕtkárová I, et al. Spinal motoneuron excitability after acute spinal cord injury in humans. Neurology 1996;47(1):231–237; doi: 10.1212/wnl.47.1.231 8710084

[86] Silver JR. Spinal shock revisited: A four-phase model. Spinal Cord 2005;43(7):450; doi: 10.1038/sj.sc.3101739 15724145

[87] Barnes CD, Joynt RJ, Schottelius BA. Motoneuron resting potentials in spinal shock. Am J Physiol 1962;203:1113–1116; doi: 10.1152/ajplegacy.1962.203.6.1113 13969356

[88] Hale LA, Society AM. The Fog of War, by Colonel Lonsdale Hale … Tuesday, 24th March, 1896. Edward Stanford, 26 and 27, Cockspur Street, Charing Cross, S.W.; 1896.

[89] Pfyffer D, Smith AC, Weber KA, et al. Prognostic value of tissue bridges in cervical spinal cord injury: A longitudinal, multicentre, retrospective cohort study. Lancet Neurol 2024;23(8):816–825; doi: 10.1016/S1474-4422(24)00173-X 38945142 PMC12128092

[90] Pfyffer D, Vallotton K, Curt A, et al. Predictive value of midsagittal tissue bridges on functional recovery after spinal cord injury. Neurorehabil Neural Repair 2021;35(1):33–43; doi: 10.1177/1545968320971787 33190619 PMC8350965

[91] Allen AR. Surgery of experimenta lesion of spinal cord equivalent to crush injury of fracture dislocation of spinal column. JAMA 1911;LVII(11):878–880.

[92] Dohrmann GJ, Wagner FC, Jr, Bucy PC. The microvasculature in transitory traumatic paraplegia. An electron microscopic study in the monkey. J Neurosurg 1971;35(3):263–271; doi: 10.3171/jns.1971.35.3.0263 22046636

[93] Bresnahan JC. An electron-microscopic analysis of axonal alterations following blunt contusion of the spinal cord of the rhesus monkey (Macaca mulatta). J Neurol Sci 1978;37(1–2):59–82; doi: 10.1016/0022-510x(78)90228-9 99494

[94] Rajaee A, Geisen ME, Sellers AK, et al. Repeat intravital imaging of the murine spinal cord reveals degenerative and reparative responses of spinal axons in real-time following a contusive SCI. Exp Neurol 2020;327:113258; doi: 10.1016/j.expneurol.2020.113258 32105708 PMC7549695

[95] Crowe MJ, Bresnahan JC, Shuman SL, et al. Apoptosis and delayed degeneration after spinal cord injury in rats and monkeys. Nat Med 1997;3(1):73–76; doi: 10.1038/nm0197-73 8986744

[96] Noble LJ, Wrathall JR. Spinal cord contusion in the rat: Morphometric analyses of alterations in the spinal cord. Exp Neurol 1985;88(1):135–149; doi: 10.1016/0014-4886(85)90119-0 3979507

[97] Blight AR. Cellular morphology of chronic spinal cord injury in the cat: Analysis of myelinated axons by line-sampling. Neuroscience 1983;10(2):521–543; doi: 10.1016/0306-4522(83)90150-1 6633870

[98] Ketelut-Carneiro N, Fitzgerald KA. Apoptosis, pyroptosis, and Necroptosis-Oh My! The many ways a cell can die. J Mol Biol 2022;434(4):167378; doi: 10.1016/j.jmb.2021.167378 34838807

[99] Shi Z, Yuan S, Shi L, et al. Programmed cell death in spinal cord injury pathogenesis and therapy. Cell Prolif 2021;54(3):e12992; doi: 10.1111/cpr.12992 33506613 PMC7941236

[100] Lu J, Ashwell KW, Waite P. Advances in secondary spinal cord injury: Role of apoptosis. Spine (Phila Pa 1976) 2000;25(14):1859–1866; doi: 10.1097/00007632-200007150-00022 10888960

[101] Liu XZ, Xu XM, Hu R, et al. Neuronal and glial apoptosis after traumatic spinal cord injury. J Neurosci 1997;17(14):5395–5406; doi: 10.1523/JNEUROSCI.17-14-05395.1997 9204923 PMC6793816

[102] Shuman SL, Bresnahan JC, Beattie MS. Apoptosis of microglia and oligodendrocytes after spinal cord contusion in rats. J Neurosci Res 1997;50(5):798–808; doi: 10.1002/(SICI)1097-4547(19971201)50:5<798::AID-JNR16>3.0.CO;2-Y 9418967

[103] Emery E, Aldana P, Bunge MB, et al. Apoptosis after traumatic human spinal cord injury. J Neurosurg 1998;89(6):911–920; doi: 10.3171/jns.1998.89.6.0911 9833815

[104] Wang SN, Guo XY, Tang J, et al. Expression and localization of absent in melanoma 2 in the injured spinal cord. Neural Regen Res 2019;14(3):542–552; doi: 10.4103/1673-5374.245481 30539825 PMC6334600

[105] Dai W, Wang X, Teng H, et al. Celastrol inhibits microglial pyroptosis and attenuates inflammatory reaction in acute spinal cord injury rats. Int Immunopharmacol 2019;66:215–223; doi: 10.1016/j.intimp.2018.11.029 30472522

[106] Zendedel A, Johann S, Mehrabi S, et al. Activation and regulation of NLRP3 Inflammasome by intrathecal application of SDF-1a in a spinal cord injury model. Mol Neurobiol 2016;53(5):3063–3075; doi: 10.1007/s12035-015-9203-5 25972240

[107] Dunai Z, Bauer PI, Mihalik R. Necroptosis: Biochemical, physiological and pathological aspects. Pathol Oncol Res 2011;17(4):791–800; doi: 10.1007/s12253-011-9433-4 21773880

[108] Fiani B, Kondilis A, Soula M, et al. Novel methods of necroptosis inhibition for spinal cord injury using translational research to limit secondary injury and enhance endogenous repair and regeneration. Neurospine 2021;18(2):261–270; doi: 10.14245/ns.2040722.361 33494555 PMC8255772

[109] Fan H, Zhang K, Shan L, et al. Reactive astrocytes undergo M1 microglia/macrohpages-induced necroptosis in spinal cord injury. Mol Neurodegener 2016;11:14; doi: 10.1186/s13024-016-0081-8 26842216 PMC4740993

[110] Liu M, Wu W, Li H, et al. Necroptosis, a novel type of programmed cell death, contributes to early neural cells damage after spinal cord injury in adult mice. J Spinal Cord Med 2015;38(6):745–753; doi: 10.1179/2045772314Y.0000000224 24970278 PMC4725808

[111] Wang Y, Wang H, Tao Y, et al. Necroptosis inhibitor necrostatin-1 promotes cell protection and physiological function in traumatic spinal cord injury. Neuroscience 2014;266:91–101; doi: 10.1016/j.neuroscience.2014.02.007 24561219

[112] Pozo Devoto VM, Lacovich V, Feole M, et al. Unraveling axonal mechanisms of traumatic brain injury. Acta Neuropathol Commun 2022;10(1):140; doi: 10.1186/s40478-022-01414-8 36131329 PMC9494812

[113] Williams PR, Marincu BN, Sorbara CD, et al. A recoverable state of axon injury persists for hours after spinal cord contusion *in vivo*. Nat Commun 2014;5:5683; doi: 10.1038/ncomms6683 25511170

[114] Nikic I, Merkler D, Sorbara C, et al. A reversible form of axon damage in experimental autoimmune encephalomyelitis and multiple sclerosis. Nat Med 2011;17(4):495–499; doi: 10.1038/nm.2324 21441916

[115] Ramón y Cajal S, DeFelipe J, Jones EG. Cajal’s Degeneration and Regeneration of the Nervous System. Oxford University Press: New York; 1991.

[116] Strich SJ. Diffuse degeneration of the cerebral white matter in severe dementia following head injury. J Neurol Neurosurg Psychiatry 1956;19(3):163–185; doi: 10.1136/jnnp.19.3.163 13357957 PMC497203

[117] Gennarelli TA, Thibault LE, Adams JH, et al. Diffuse axonal injury and traumatic coma in the primate. Ann Neurol 1982;12(6):564–574; doi: 10.1002/ana.410120611 7159060

[118] Adams JH, Graham DI, Murray LS, et al. Diffuse axonal injury due to nonmissile head injury in humans: An analysis of 45 cases. Ann Neurol 1982;12(6):557–563; doi: 10.1002/ana.410120610 7159059

[119] Erb DE, Povlishock JT. Axonal damage in severe traumatic brain injury: An experimental study in cat. Acta Neuropathol 1988;76(4):347–358; doi: 10.1007/BF00686971 2459896

[120] Hellewell SC, Yan EB, Agyapomaa DA, et al. Post-traumatic hypoxia exacerbates brain tissue damage: Analysis of axonal injury and glial responses. J Neurotrauma 2010;27(11):1997–2010; doi: 10.1089/neu.2009.1245 20822466

[121] LoPachin RM, Gaughan CL, Lehning EJ, et al. Experimental spinal cord injury: Spatiotemporal characterization of elemental concentrations and water contents in axons and neuroglia. J Neurophysiol 1999;82(5):2143–2153; doi: 10.1152/jn.1999.82.5.2143 10561394

[122] Pettus EH, Christman CW, Giebel ML, et al. Traumatically induced altered membrane permeability: Its relationship to traumatically induced reactive axonal change. J Neurotrauma 1994;11(5):507–522; doi: 10.1089/neu.1994.11.507 7861444

[123] Povlishock JT, Pettus EH. Traumatically induced axonal damage: Evidence for enduring changes in axolemmal permeability with associated cytoskeletal change. Acta Neurochir (Suppl 1996);66:81–86; doi: 10.1007/978-3-7091-9465-2_15 8780803

[124] Kilinc D, Gallo G, Barbee KA. Mechanical membrane injury induces axonal beading through localized activation of calpain. Exp Neurol 2009;219(2):553–561; doi: 10.1016/j.expneurol.2009.07.014 19619536 PMC2747288

[125] Wolf JA, Stys PK, Lusardi T, et al. Traumatic axonal injury induces calcium influx modulated by tetrodotoxin-sensitive sodium channels. J Neurosci 2001;21(6):1923–1930; doi: 10.1523/JNEUROSCI.21-06-01923.2001 11245677 PMC6762603

[126] Smith DH, Wolf JA, Lusardi TA, et al. High tolerance and delayed elastic response of cultured axons to dynamic stretch injury. J Neurosci 1999;19(11):4263–4269; doi: 10.1523/JNEUROSCI.19-11-04263.1999 10341230 PMC6782601

[127] LoPachin RM, Jr, Stys PK. Elemental composition and water content of rat optic nerve myelinated axons and glial cells: Effects of *in vitro* anoxia and reoxygenation. J Neurosci 1995;15(10):6735–6746; doi: 10.1523/JNEUROSCI.15-10-06735.1995 7472432 PMC6578013

[128] Stys PK, Lopachin RM, Jr, Elemental composition and water content of rat optic nerve myelinated axons during *in vitro* post-anoxia reoxygenation. Neuroscience 1996;73(4):1081–1090; doi: 10.1016/0306-4522(96)00114-5 8809826

[129] Shcherbatko A, Ono F, Mandel G, et al. Voltage-dependent sodium channel function is regulated through membrane mechanics. Biophys J 1999;77(4):1945–1959; doi: 10.1016/S0006-3495(99)77036-0 10512815 PMC1300476

[130] Tabarean IV, Juranka P, Morris CE. Membrane stretch affects gating modes of a skeletal muscle sodium channel. Biophys J 1999;77(2):758–774; doi: 10.1016/S0006-3495(99)76930-4 10423424 PMC1300370

[131] Nikolaeva MA, Mukherjee B, Stys PK. Na+-dependent sources of intra-axonal Ca2+ release in rat optic nerve during *in vitro* chemical ischemia. J Neurosci 2005;25(43):9960–9967; doi: 10.1523/JNEUROSCI.2003-05.2005 16251444 PMC6725557

[132] Iwata A, Stys PK, Wolf JA, et al. Traumatic axonal injury induces proteolytic cleavage of the voltage-gated sodium channels modulated by tetrodotoxin and protease inhibitors. J Neurosci 2004;24(19):4605–4613; doi: 10.1523/JNEUROSCI.0515-03.2004 15140932 PMC6729402

[133] Stys PK, Waxman SG, Ransom BR. Na(+)-Ca2+ exchanger mediates Ca2+ influx during anoxia in mammalian central nervous system white matter. Ann Neurol 1991;30(3):375–380; doi: 10.1002/ana.410300309 1952825

[134] von Reyn CR, Spaethling JM, Mesfin MN, et al. Calpain mediates proteolysis of the voltage-gated sodium channel alpha-subunit. J Neurosci 2009;29(33):10350–10356; doi: 10.1523/JNEUROSCI.2339-09.2009 19692609 PMC2799681

[135] Staal JA, Dickson TC, Gasperini R, et al. Initial calcium release from intracellular stores followed by calcium dysregulation is linked to secondary axotomy following transient axonal stretch injury. J Neurochem 2010;112(5):1147–1155; doi: 10.1111/j.1471-4159.2009.06531.x 19968758

[136] He XS, Xiang Z, Zhou F, et al. Calcium overloading in traumatic axonal injury by lateral head rotation: A morphological evidence in rat model. J Clin Neurosci 2004;11(4):402–407; doi: 10.1016/j.jocn.2004.01.001 15080957

[137] Young W, Yen V, Blight A. Extracellular calcium ionic activity in experimental spinal cord contusion. Brain Res 1982;253(1–2):105–113; doi: 10.1016/0006-8993(82)90677-1 6295546

[138] Steffensen I, Waxman SG, Mills L, et al. Immunolocalization of the Na(+)-Ca2+ exchanger in mammalian myelinated axons. Brain Res 1997;776(1–2):1–9; doi: 10.1016/s0006-8993(97)00868-8 9439790

[139] Stys PK, Waxman SG, Ransom BR. Ionic mechanisms of anoxic injury in mammalian CNS white matter: Role of Na+ channels and Na(+)-Ca2+ exchanger. J Neurosci 1992;12(2):430–439; doi: 10.1523/JNEUROSCI.12-02-00430.1992 1311030 PMC6575619

[140] Barsukova AG, Forte M, Bourdette D. Focal increases of axoplasmic Ca2+, aggregation of sodium-calcium exchanger, N-type Ca2+ channel, and actin define the sites of spheroids in axons undergoing oxidative stress. J Neurosci 2012;32(35):12028–12037; doi: 10.1523/JNEUROSCI.0408-12.2012 22933787 PMC3484163

[141] Omelchenko A, Shrirao AB, Bhattiprolu AK, et al. Dynamin and reverse-mode sodium calcium exchanger blockade confers neuroprotection from diffuse axonal injury. Cell Death Dis 2019;10(10):727; doi: 10.1038/s41419-019-1908-3 31562294 PMC6765020

[142] Fern R, Ransom BR, Stys PK, et al. Pharmacological protection of CNS white matter during anoxia: Actions of phenytoin, carbamazepine and diazepam. J Pharmacol Exp Ther 1993;266(3):1549–1555. 8371157

[143] Imaizumi T, Kocsis JD, Waxman SG. The role of voltage-gated Ca2+ channels in anoxic injury of spinal cord white matter. Brain Res 1999;817(1–2):84–92; doi: 10.1016/s0006-8993(98)01214-1 9889329

[144] von Reyn CR, Mott RE, Siman R, et al. Mechanisms of calpain mediated proteolysis of voltage gated sodium channel alpha-subunits following *in vitro* dynamic stretch injury. J Neurochem 2012;121(5):793–805; doi: 10.1111/j.1471-4159.2012.07735.x 22428606 PMC4946955

[145] Stirling DP, Stys PK. Mechanisms of axonal injury: Internodal nanocomplexes and calcium deregulation. Trends Mol Med 2010;16(4):160–170; doi: 10.1016/j.molmed.2010.02.002 20207196 PMC2976657

[146] Stirling DP, Cummins K, Wayne Chen SR, et al. Axoplasmic reticulum Ca(2+) release causes secondary degeneration of spinal axons. Ann Neurol 2014;75(2):220–229; doi: 10.1002/ana.24099 24395428

[147] Orem BC, Pelisch N, Williams J, et al. Intracellular calcium release through IP(3)R or RyR contributes to secondary axonal degeneration. Neurobiol Dis 2017;106:235–243; doi: 10.1016/j.nbd.2017.07.011 28709993

[148] Orem BC, Rajaee A, Stirling DP. Inhibiting calcium release from ryanodine receptors protects axons after spinal cord injury. J Neurotrauma 2022;39(3–4):311–319; doi: 10.1089/neu.2021.0350 34913747 PMC8817717

[149] Orem BC, Partain SB, Stirling DP. Inhibiting store-operated calcium entry attenuates white matter secondary degeneration following SCI. Neurobiol Dis 2020;136:104718; doi: 10.1016/j.nbd.2019.104718 31846736

[150] Orem BC, Morehouse JR, Ames S, et al. Direct Ryanodine receptor-2 knockout in primary afferent fibers modestly affects neurological recovery after contusive spinal cord injury. Neurotrauma Rep 2022;3(1):433–446; doi: 10.1089/neur.2022.0044 36337076 PMC9622210

[151] Dumont RJ, Okonkwo DO, Verma S, et al. Acute spinal cord injury, part I: Pathophysiologic mechanisms. Clin Neuropharmacol 2001;24(5):254–264; doi: 10.1097/00002826-200109000-00002 11586110

[152] Munteanu C, Rotariu M, Turnea M, et al. Main cations and cellular biology of traumatic spinal cord injury. Cells 2022;11(16); doi: 10.3390/cells11162503 PMC9406880 36010579

[153] Halestrap AP. What is the mitochondrial permeability transition pore? J Mol Cell Cardiol 2009;46(6):821–831; doi: 10.1016/j.yjmcc.2009.02.021 19265700

[154] Crompton M, Costi A. Kinetic evidence for a heart mitochondrial pore activated by Ca2+, inorganic phosphate and oxidative stress. A potential mechanism for mitochondrial dysfunction during cellular Ca2+ overload. Eur J Biochem 1988;178(2):489–501; doi: 10.1111/j.1432-1033.1988.tb14475.x 2850179

[155] Barrientos SA, Martinez NW, Yoo S, et al. Axonal degeneration is mediated by the mitochondrial permeability transition pore. J Neurosci 2011;31(3):966–978; doi: 10.1523/JNEUROSCI.4065-10.2011 21248121 PMC3245862

[156] Buki A, Okonkwo DO, Wang KK, et al. Cytochrome c release and caspase activation in traumatic axonal injury. J Neurosci 2000;20(8):2825–2834; doi: 10.1523/JNEUROSCI.20-08-02825.2000 10751434 PMC6772193

[157] Ma M. Role of calpains in the injury-induced dysfunction and degeneration of the mammalian axon. Neurobiol Dis 2013;60:61–79; doi: 10.1016/j.nbd.2013.08.010 23969238 PMC3882011

[158] Saatman KE, Abai B, Grosvenor A, et al. Traumatic axonal injury results in biphasic calpain activation and retrograde transport impairment in mice. J Cereb Blood Flow Metab 2003;23(1):34–42; doi: 10.1097/01.WCB.0000035040.10031.B0 12500089

[159] Saatman KE, Bozyczko-Coyne D, Marcy V, et al. Prolonged calpain-mediated spectrin breakdown occurs regionally following experimental brain injury in the rat. J Neuropathol Exp Neurol 1996;55(7):850–860; doi: 10.1097/00005072-199607000-00010 8965100

[160] George EB, Glass JD, Griffin JW. Axotomy-induced axonal degeneration is mediated by calcium influx through ion-specific channels. J Neurosci 1995;15(10):6445–6452; doi: 10.1523/JNEUROSCI.15-10-06445.1995 7472407 PMC6577979

[161] Buki A, Siman R, Trojanowski JQ, et al. The role of calpain-mediated spectrin proteolysis in traumatically induced axonal injury. J Neuropathol Exp Neurol 1999;58(4):365–375; doi: 10.1097/00005072-199904000-00007 10218632

[162] Ray SK, Hogan EL, Banik NL. Calpain in the pathophysiology of spinal cord injury: Neuroprotection with calpain inhibitors. Brain Res Brain Res Rev 2003;42(2):169–185; doi: 10.1016/s0165-0173(03)00152-8 12738057

[163] Wingrave JM, Schaecher KE, Sribnick EA, et al. Early induction of secondary injury factors causing activation of calpain and mitochondria-mediated neuronal apoptosis following spinal cord injury in rats. J Neurosci Res 2003;73(1):95–104; doi: 10.1002/jnr.10607 12815713

[164] Yang J, Weimer RM, Kallop D, et al. Regulation of axon degeneration after injury and in development by the endogenous calpain inhibitor calpastatin. Neuron 2013;80(5):1175–1189; doi: 10.1016/j.neuron.2013.08.034 24210906

[165] Khan M, Dhammu TS, Singh I, et al. Amelioration of spinal cord injury in rats by blocking peroxynitrite/calpain activity. BMC Neurosci 2018;19(1):50; doi: 10.1186/s12868-018-0450-z 30103682 PMC6090709

[166] Wang Y, Liu Y, Lopez D, et al. Protection against TBI-Induced neuronal death with post-treatment with a selective Calpain-2 inhibitor in mice. J Neurotrauma 2018;35(1):105–117; doi: 10.1089/neu.2017.5024 28594313 PMC5757088

[167] Povlishock JT, Becker DP, Cheng CL, et al. Axonal change in minor head injury. J Neuropathol Exp Neurol 1983;42(3):225–242; doi: 10.1097/00005072-198305000-00002 6188807

[168] Christman CW, Grady MS, Walker SA, et al. Ultrastructural studies of diffuse axonal injury in humans. J Neurotrauma 1994;11(2):173–186; doi: 10.1089/neu.1994.11.173 7523685

[169] Wang J, Hamm RJ, Povlishock JT. Traumatic axonal injury in the optic nerve: Evidence for axonal swelling, disconnection, dieback, and reorganization. J Neurotrauma 2011;28(7):1185–1198; doi: 10.1089/neu.2011.1756 21506725 PMC3136743

[170] Tang-Schomer MD, Johnson VE, Baas PW, et al. Partial interruption of axonal transport due to microtubule breakage accounts for the formation of periodic varicosities after traumatic axonal injury. Exp Neurol 2012;233(1):364–372; doi: 10.1016/j.expneurol.2011.10.030 22079153 PMC3979336

[171] Gennarelli TA, Thibault LE, Tipperman R, et al. Axonal injury in the optic nerve: A model simulating diffuse axonal injury in the brain. J Neurosurg 1989;71(2):244–253; doi: 10.3171/jns.1989.71.2.0244 2746348

[172] Maxwell WL, Irvine A, Adams JH, et al. Focal axonal injury: The early axonal response to stretch. J Neurocytol 1991;20(3):157–164; doi: 10.1007/BF01186989 1709964

[173] Chen XH, Siman R, Iwata A, et al. Long-term accumulation of amyloid-beta, beta-secretase, presenilin-1, and caspase-3 in damaged axons following brain trauma. Am J Pathol 2004;165(2):357–371; doi: 10.1016/s0002-9440(10)63303-2 15277212 PMC1618579

[174] Maxwell WL, Bartlett E, Morgan H. Wallerian degeneration in the optic nerve stretch-injury model of traumatic brain injury: A stereological analysis. J Neurotrauma 2015;32(11):780–790; doi: 10.1089/neu.2014.3369 25333317

[175] Wilhelmy B, Gerzanich V, Simard JM, et al. The NCX1 calcium exchanger is implicated in delayed axotomy after peripheral nerve stretch injury. J Peripher Nerv Syst 2024;29(4):555–566; doi: 10.1111/jns.12663 39402795

[176] Blumbergs PC, Scott G, Manavis J, et al. Topography of axonal injury as defined by amyloid precursor protein and the sector scoring method in mild and severe closed head injury. J Neurotrauma 1995;12(4):565–572; doi: 10.1089/neu.1995.12.565 8683607

[177] Sherriff FE, Bridges LR, Gentleman SM, et al. Markers of axonal injury in post mortem human brain. Acta Neuropathol 1994;88(5):433–439; doi: 10.1007/BF00389495 7847072

[178] Li GL, Farooque M, Holtz A, et al. Changes of beta-amyloid precursor protein after compression trauma to the spinal cord: An experimental study in the rat using immunohistochemistry. J Neurotrauma 1995;12(3):269–277; doi: 10.1089/neu.1995.12.269 7473801

[179] Hanell A, Greer JE, McGinn MJ, et al. Traumatic brain injury-induced axonal phenotypes react differently to treatment. Acta Neuropathol 2015;129(2):317–332; doi: 10.1007/s00401-014-1376-x 25528329

[180] Johnson VE, Stewart W, Weber MT, et al. SNTF immunostaining reveals previously undetected axonal pathology in traumatic brain injury. Acta Neuropathol 2016;131(1):115–135; doi: 10.1007/s00401-015-1506-0 26589592 PMC4780426

[181] Greer JE, McGinn MJ, Povlishock JT. Diffuse traumatic axonal injury in the mouse induces atrophy, c-Jun activation, and axonal outgrowth in the axotomized neuronal population. J Neurosci 2011;31(13):5089–5105; doi: 10.1523/JNEUROSCI.5103-10.2011 21451046 PMC3076099

[182] Baker AJ, Phan N, Moulton RJ, et al. Attenuation of the electrophysiological function of the corpus callosum after fluid percussion injury in the rat. J Neurotrauma 2002;19(5):587–599; doi: 10.1089/089771502753754064 12042094

[183] Weber MT, Arena JD, Xiao R, et al. CLARITY reveals a more protracted temporal course of axon swelling and disconnection than previously described following traumatic brain injury. Brain Pathol 2019;29(3):437–450; doi: 10.1111/bpa.12677 30444552 PMC6482960

[184] Tator CH, Fehlings MG. Review of the secondary injury theory of acute spinal cord trauma with emphasis on vascular mechanisms. J Neurosurg 1991;75(1):15–26; doi: 10.3171/jns.1991.75.1.0015 2045903

[185] Fairholm DJ, Turnbull IM. Microangiographic study of experimental spinal cord injuries. J Neurosurg 1971;35(3):277–286; doi: 10.3171/jns.1971.35.3.0277 22046638

[186] Fried LC, Goodkin R. Microangiographic observations of the experimentally traumatized spinal cord. J Neurosurg 1971;35(6):709–714; doi: 10.3171/jns.1971.35.6.0709 5000663

[187] Ducker TB, Perot PL, Jr, Local tissue oxygen and blood flow in the acutely injured spinal cord. Proc Veterans Adm Spinal Cord Inj Conf 1971;18:29–32. 5161642

[188] Griffiths IR. Spinal cord blood flow after acute experimental cord injury in dogs. J Neurol Sci 1976;27(2):247–259; doi: 10.1016/0022-510x(76)90065-4 1249588

[189] Ishige N, Pitts LH, Hashimoto T, et al. Effect of hypoxia on traumatic brain injury in rats: Part 1. Changes in neurological function, electroencephalograms, and histopathology. Neurosurgery 1987;20(6):848–853; doi: 10.1227/00006123-198706000-00005 3614563

[190] Badhiwala JH, Wilson JR, Witiw CD, et al. The influence of timing of surgical decompression for acute spinal cord injury: A pooled analysis of individual patient data. Lancet Neurol 2021;20(2):117–126; doi: 10.1016/S1474-4422(20)30406-3 33357514

[191] Fehlings MG, Hachem LD, Tetreault LA, et al. Timing of decompressive surgery in patients with acute spinal cord injury: Systematic review update. Global Spine J 2024;14(3_suppl):38S–57S; doi: 10.1177/21925682231197404 38526929 PMC10964893

[192] Aarabi B, Akhtar-Danesh N, Simard JM, et al. Efficacy of Early (</= 24 Hours), Late (25-72 Hours), and delayed (>72 Hours) surgery with magnetic resonance imaging-confirmed decompression in American Spinal Injury Association impairment scale grades C and D acute traumatic central cord syndrome caused by spinal stenosis. J Neurotrauma 2021;38(15):2073–2083; doi: 10.1089/neu.2021.0040 33726507 PMC8309437

[193] Aarabi B, Olexa J, Chryssikos T, et al. Extent of spinal cord decompression in motor complete (American Spinal Injury Association Impairment Scale Grades A and B) traumatic spinal cord injury patients: Post-Operative magnetic resonance imaging analysis of standard operative approaches. J Neurotrauma 2019;36(6):862–876; doi: 10.1089/neu.2018.5834 30215287 PMC6484360

[194] Donnelly DJ, Popovich PG. Inflammation and its role in neuroprotection, axonal regeneration and functional recovery after spinal cord injury. Exp Neurol 2008;209(2):378–388; doi: 10.1016/j.expneurol.2007.06.009 17662717 PMC2692462

[195] Campbell SJ, Wilcockson DC, Butchart AG, et al. Altered chemokine expression in the spinal cord and brain contributes to differential interleukin-1beta-induced neutrophil recruitment. J Neurochem 2002;83(2):432–441; doi: 10.1046/j.1471-4159.2002.01166.x 12423253

[196] Schnell L, Fearn S, Klassen H, et al. Acute inflammatory responses to mechanical lesions in the CNS: Differences between brain and spinal cord. Eur J Neurosci 1999;11(10):3648–3658; doi: 10.1046/j.1460-9568.1999.00792.x 10564372

[197] Schnell L, Fearn S, Schwab ME, et al. Cytokine-induced acute inflammation in the brain and spinal cord. J Neuropathol Exp Neurol 1999;58(3):245–254; doi: 10.1097/00005072-199903000-00004 10197816

[198] Kubota K, Saiwai H, Kumamaru H, et al. Myeloperoxidase exacerbates secondary injury by generating highly reactive oxygen species and mediating neutrophil recruitment in experimental spinal cord injury. Spine (Phila Pa 1976) 2012;37(16):1363–1369; doi: 10.1097/BRS.0b013e31824b9e77 22322369

[199] Brinkmann V, Reichard U, Goosmann C, et al. Neutrophil extracellular traps kill bacteria. Science 2004;303(5663):1532–1535; doi: 10.1126/science.1092385 15001782

[200] Feng Z, Min L, Liang L, et al. Neutrophil extracellular traps exacerbate secondary injury via promoting Neuroinflammation and blood-spinal cord barrier disruption in spinal cord injury. Front Immunol 2021;12:698249; doi: 10.3389/fimmu.2021.698249 34456910 PMC8385494

[201] Gensel JC, Zhang B. Macrophage activation and its role in repair and pathology after spinal cord injury. Brain Res 2015;1619:1–11; doi: 10.1016/j.brainres.2014.12.045 25578260

[202] Kigerl KA, Gensel JC, Ankeny DP, et al. Identification of two distinct macrophage subsets with divergent effects causing either neurotoxicity or regeneration in the injured mouse spinal cord. J Neurosci 2009;29(43):13435–13444; doi: 10.1523/JNEUROSCI.3257-09.2009 19864556 PMC2788152

[203] Conti A, Miscusi M, Cardali S, et al. Nitric oxide in the injured spinal cord: Synthases cross-talk, oxidative stress and inflammation. Brain Res Rev 2007;54(1):205–218; doi: 10.1016/j.brainresrev.2007.01.013 17500094

[204] Okada S, Nakamura M, Mikami Y, et al. Blockade of interleukin-6 receptor suppresses reactive astrogliosis and ameliorates functional recovery in experimental spinal cord injury. J Neurosci Res 2004;76(2):265–276; doi: 10.1002/jnr.20044 15048924

[205] Bermudez S, Khayrullina G, Zhao Y, et al. NADPH oxidase isoform expression is temporally regulated and may contribute to microglial/macrophage polarization after spinal cord injury. Mol Cell Neurosci 2016;77:53–64; doi: 10.1016/j.mcn.2016.10.001 27729244 PMC5124517

[206] Satake K, Matsuyama Y, Kamiya M, et al. Nitric oxide via macrophage iNOS induces apoptosis following traumatic spinal cord injury. Brain Res Mol Brain Res 2000;85(1–2):114–122; doi: 10.1016/s0169-328x(00)00253-9 11146113

[207] Evans TA, Barkauskas DS, Myers JT, et al. High-resolution intravital imaging reveals that blood-derived macrophages but not resident microglia facilitate secondary axonal dieback in traumatic spinal cord injury. Exp Neurol 2014;254:109–120; doi: 10.1016/j.expneurol.2014.01.013 24468477 PMC3954731

[208] McPhail LT, Stirling DP, Tetzlaff W, et al. The contribution of activated phagocytes and myelin degeneration to axonal retraction/dieback following spinal cord injury. Eur J Neurosci 2004;20(8):1984–1994; doi: 10.1111/j.1460-9568.2004.03662.x 15450077

[209] Bracken MB, Shepard MJ, Collins WF, et al. A randomized, controlled trial of methylprednisolone or naloxone in the treatment of acute spinal-cord injury. Results of the Second National Acute Spinal Cord Injury Study. N Engl J Med 1990;322(20):1405–1411; doi: 10.1056/NEJM199005173222001 2278545

[210] Bracken MB, Shepard MJ, Holford TR, et al. Administration of methylprednisolone for 24 or 48 hours or tirilazad mesylate for 48 hours in the treatment of acute spinal cord injury. Results of the Third National Acute Spinal Cord Injury Randomized Controlled Trial. National Acute Spinal Cord Injury Study. JAMA 1997;277(20):1597–1604. 9168289

[211] Bilgen M, Abbe R, Liu SJ, et al. Spatial and temporal evolution of hemorrhage in the hyperacute phase of experimental spinal cord injury: *In vivo* magnetic resonance imaging. Magn Reson Med 2000;43(4):594–600; doi: 10.1002/(sici)1522-2594(200004)43:4<594::aid-mrm15>3.0.co;2-1 10748436

[212] Gerzanich V, Woo SK, Vennekens R, et al. De novo expression of Trpm4 initiates secondary hemorrhage in spinal cord injury. Nat Med 2009;15(2):185–191; doi: 10.1038/nm.1899 19169264 PMC2730968

[213] Wagner FC, Jr, Stewart WB. Effect of trauma dose on spinal cord edema. J Neurosurg 1981;54(6):802–806; doi: 10.3171/jns.1981.54.6.0802 6165810

[214] Leonard AV, Thornton E, Vink R. The relative contribution of edema and hemorrhage to raised intrathecal pressure after traumatic spinal cord injury. J Neurotrauma 2015;32(6):397–402; doi: 10.1089/neu.2014.3543 25111333

[215] Yashon D, Bingham WG, Jr, Faddoul EM, et al. Edema of the spinal cord following experimental impact trauma. J Neurosurg 1973;38(6):693–697; doi: 10.3171/jns.1973.38.6.0693 4197015

[216] Seblani M, Decherchi P, Brezun JM. Edema after CNS trauma: A focus on spinal cord injury. Int J Mol Sci 2023;24(8); doi: 10.3390/ijms24087159 PMC10138956 37108324

[217] Stokum JA, Gerzanich V, Simard JM. Molecular pathophysiology of cerebral edema. J Cereb Blood Flow Metab 2016;36(3):513–538; doi: 10.1177/0271678X15617172 26661240 PMC4776312

[218] Aarabi B, Simard JM, Kufera JA, et al. Intramedullary lesion expansion on magnetic resonance imaging in patients with motor complete cervical spinal cord injury. J Neurosurg Spine 2012;17(3):243–250; doi: 10.3171/2012.6.SPINE12122 22794535 PMC3534760

[219] Le E, Aarabi B, Hersh DS, et al. Predictors of intramedullary lesion expansion rate on MR images of patients with subaxial spinal cord injury. J Neurosurg Spine 2015;22(6):611–621; doi: 10.3171/2014.10.SPINE14576 25746115

[220] Stokum JA, Cannarsa GJ, Wessell AP, et al. When the blood hits your brain: The neurotoxicity of Extravasated blood. Int J Mol Sci 2021;22(10); doi: 10.3390/ijms22105132 PMC8151992 34066240

[221] Losey P, Young C, Krimholtz E, et al. The role of hemorrhage following spinal-cord injury. Brain Res 2014;1569:9–18; doi: 10.1016/j.brainres.2014.04.033 24792308

